# The continuum disordered pinning model

**DOI:** 10.1007/s00440-014-0606-4

**Published:** 2014-12-17

**Authors:** Francesco Caravenna, Rongfeng Sun, Nikos Zygouras

**Affiliations:** 1 Dipartimento di Matematica e Applicazioni, Università degli Studi di Milano-Bicocca, via Cozzi 55, 20125 Milano, Italy; 2 Department of Mathematics, National University of Singapore, 10 Lower Kent Ridge Road, Singapore, 119076 Singapore; 3 Department of Statistics, University of Warwick, Coventry, CV4 7AL UK

**Keywords:** Scaling limit, Disorder relevance, Weak disorder, Pinning model, Fell–Matheron topology, Hausdorff metric, Random polymer, Wiener Chaos expansion, Primary 82B44, Secondary 82D60, 60K35

## Abstract

Any renewal processes on ${\mathbb {N}}_0$ with a polynomial tail, with exponent $\alpha \in (0,1)$, has a non-trivial scaling limit, known as the $\alpha$-*stable regenerative set*. In this paper we consider Gibbs transformations of such renewal processes in an i.i.d. random environment, called disordered pinning models. We show that for $\alpha \in \left( \frac{1}{2}, 1\right)$ these models have a universal scaling limit, which we call the *continuum disordered pinning model* (CDPM). This is a random closed subset of ${\mathbb {R}}$ in a white noise random environment, with subtle features:

- Any *fixed* a.s. property of the $\alpha$-stable regenerative set (e.g., its Hausdorff dimension) is also an a.s. property of the CDPM, for almost every realization of the environment.
- Nonetheless, the law of the CDPM is *singular* with respect to the law of the $\alpha$-stable regenerative set, for almost every realization of the environment.

The existence of a disordered continuum model, such as the CDPM, is a manifestation of *disorder relevance* for pinning models with $\alpha \in \left( \frac{1}{2}, 1\right)$.

Any *fixed* a.s. property of the $\alpha$-stable regenerative set (e.g., its Hausdorff dimension) is also an a.s. property of the CDPM, for almost every realization of the environment.

Nonetheless, the law of the CDPM is *singular* with respect to the law of the $\alpha$-stable regenerative set, for almost every realization of the environment.

## Introduction

We consider disordered pinning models, which are defined via a Gibbs change of measure of a renewal process, depending on an external i.i.d. random environment. First introduced in the physics and biology literature, these models have attracted much attention due to their rich structure, which is amenable to a rigorous investigation; see, e.g., the monographs of Giacomin [19, 20] and den Hollander [13].

In this paper we define a *continuum disordered pinning model* (CDPM), inspired by recent work of Alberts et al. [4] on the directed polymer in random environment. The interest for such a continuum model is manifold:

- It is a *universal object*, arising as the scaling limit of discrete disordered pinning models in a suitable continuum and weak disorder limit, Theorem 1.3.
- It provides a tool to capture the emerging effect of disorder in pinning models, when disorder is relevant, Sect. 1.4 for a more detailed discussion.
- It can be interpreted as an $\alpha$-stable regenerative set in a white noise random environment, displaying subtle properties, Theorems 1.4, 1.5 and 1.6.

Throughout the paper, we use the conventions ${\mathbb {N}}:= \{1,2,\ldots \}$, ${\mathbb N} _0:=\{0\}\cup {\mathbb {N}}$, and write $a_n \sim b_n$ to mean $\lim _{n\rightarrow \infty } a_n/b_n = 1$.

### Renewal processes and regenerative sets

Let $\tau := (\tau _n)_{n\ge 0}$ be a *renewal process* on ${\mathbb {N}}_0$, that is $\tau _0 = 0$ and the increments $(\tau _n-\tau _{n-1})_{n\in {\mathbb {N}}}$ are i.i.d. ${\mathbb {N}}$-valued random variables (so that $0 = \tau _0 < \tau _1 < \tau _2 < \cdots$). Probability and expectation for $\tau$ will be denoted respectively by $\mathrm P$ and $\mathrm E$. We assume that $\tau$ is *non-terminating*, i.e., $\mathrm P(\tau _1 < \infty ) = 1$, and

1.1 $$\begin{aligned} K(n):=\mathrm P(\tau _1=n) = \frac{L(n)}{n^{1+\alpha }} , \qquad \text {as } n\rightarrow \infty , \end{aligned}$$

where $\alpha \in (0,1)$ and $L(\cdot )$ is a slowly varying function [8]. We assume for simplicity that $K(n)>0$ for every $n\in {\mathbb N}$ (periodicity complicates notation, but can be easily incorporated).

Let us denote by ${\mathcal C}$ the space of all *closed subsets of* ${\mathbb {R}}$. There is a natural topology on ${\mathcal C}$, called the Fell–Matheron topology [15, 24, 25], which turns ${\mathcal C}$ into a *compact Polish space*, i.e. a compact separable topological space which admits a complete metric. This can be taken as a version of the *Hausdorff distance* (see Appendix  for more details).

Identifying the renewal process $\tau =\{\tau _n\}_{n\ge 0}$ with its range, we may view $\tau$ as a random subset of ${\mathbb {N}}_0$, i.e. as a ${\mathcal C}$-value random variable (hence we write $\{n \in \tau \} := \bigcup _{k\ge 0} \{\tau _k = n\}$). This viewpoint is very fruitful, because as $N\rightarrow \infty$ the rescaled set

1.2 $$\begin{aligned} \frac{\tau }{N} = \bigg \{ \frac{\tau _n}{N} \bigg \}_{n\ge 0} \end{aligned}$$

converges in distribution on ${\mathcal C}$ to a universal random closed set $\varvec{\tau }$ of $[0,\infty )$, called the $\alpha$-*stable regenerative set* ([16], [19, Thm. A.8]). This coincides with the closure of the range of the $\alpha$-stable subordinator or, equivalently, with the zero level set of a Bessel process of dimension $\delta = 2(1-\alpha )$ (see Appendix ), and we denote its law by $\varvec{\mathrm {P}}^\alpha$.

#### *Remark 1.1*

Random sets have been studied extensively [24, 25]. Here we focus on the special case of random closed subsets of ${\mathbb {R}}$. The theory developed in [16] for *regenerative sets* cannot be applied in our context, because we modify renewal processes through inhomogeneous perturbations and conditioning (see (1.4)–(1.9) below). For this reason, in Appendix  we review and develop a general framework to study convergence of random closed sets of ${\mathbb {R}}$, based on a natural notion of finite-dimensional distributions.

### Disordered pinning models

Let $\omega :=(\omega _n)_{n\in {\mathbb {N}}}$ be i.i.d. random variables (independent of the renewal process $\tau$), which represent the disorder. Probability and expectation for $\omega$ will be denoted respectively by ${\mathbb P}$ and ${\mathbb E}$. We assume that

1.3 $$\begin{aligned} {\mathbb E} [\omega _n]=0, \qquad {{\mathrm{\mathbb {V}ar}}}(\omega _n)=1, \qquad \exists t_0 > 0: \ \ \Lambda (t):=\log {\mathbb E} [e^{t \omega _n} ]<\infty \ \ \text {for}\, |t| \le t_0. \end{aligned}$$

The *disordered pinning model* is a random probability law $\mathrm P^{\omega }_{N,\beta ,h}$ on subsets of $\{0, \ldots , N\}$, indexed by realizations $\omega$ of the disorder, the system size $N\in {\mathbb {N}}$, the disorder strength $\beta >0$ and bias $h\in {\mathbb {R}}$, defined by the following Gibbs change of measure of the renewal process $\tau$:

1.4 $$\begin{aligned} \frac{\mathrm P^{\omega }_{N,\beta ,h} (\tau \cap [0,N])}{\mathrm P(\tau \cap [0,N])} :=\frac{1}{Z^{\omega }_{N,\beta ,h}} e^{\sum _{n=1}^N (\beta \omega _n -\Lambda (\beta ) + h) 1\!\!1_{\{n\in \tau \}}}, \end{aligned}$$

where the normalizing constant

1.5 $$\begin{aligned} Z_{N,\beta ,h}^{\omega }:=\mathrm E\Big [ e^{\sum _{n=1}^N (\beta \omega _n -\Lambda (\beta ) + h) 1\!\!1_{\{n\in \tau \}}} \Big ] \end{aligned}$$

is called the *partition function*. In words, we perturb the law of the renewal process $\tau$ in the interval $[0,N]$, by giving rewards/penalties $(\beta \omega _n - \Lambda (\beta ) + h)$ to each visited site $n \in \tau$. (The presence of the factor $\Lambda (\beta )$ in (1.4)–(1.5), which just corresponds to a translation of $h$, allows to have normalized weights ${\mathbb E} [e^{\beta \omega _n - \Lambda (\beta )}] = 1$ for $h=0$).

The properties of the model $\mathrm P^{\omega }_{N,\beta ,h}$, especially in the limit $N\rightarrow \infty$, have been studied in depth in the recent mathematical literature (see e.g. [13, 19, 20] for an overview). In this paper we focus on the problem of defining a continuum analogue of $\mathrm P^{\omega }_{N,\beta ,h}$.

Since under the “free law” $\mathrm P$ the rescaled renewal process $\tau / N$ converges in distribution to the $\alpha$-stable regenerative set $\varvec{\tau }$, it is natural to ask what happens under the “interacting law” $\mathrm P^{\omega }_{N,\beta ,h}$. Heuristically, in the scaling limit the i.i.d. random variables $(\omega _n)_{n\in {\mathbb {N}}}$ should be replaced by a one-dimensional white noise $(\mathrm {d}W_t)_{t \in [0,\infty )}$, where $W = (W_t)_{t\in [0,\infty )}$ denotes a standard Brownian motion (independent of $\varvec{\tau }$). Looking at (1.4), a natural candidate for the scaling limit of $\tau /N$ under $\mathrm P^{\omega }_{N,\beta ,h}$ would be the random measure $\varvec{\mathrm {P}}^{\alpha ;W}_{T,\beta , h}$ on ${\mathcal C}$ defined by

1.6 $$\begin{aligned} \frac{\mathrm {d}\varvec{\mathrm {P}}^{\alpha ;W}_{T, \beta , h}}{\mathrm {d}\varvec{\mathrm {P}}^\alpha }(\varvec{\tau }\cap [0,T]) := \frac{1}{\varvec{Z}^{\alpha ; W}_{T, \beta , h}} e^{\int _0^T 1\!\!1_{\{t\in \varvec{\tau }\}} \left( \beta \mathrm {d}W_t + \left( h-\frac{1}{2}\beta ^2\right) \mathrm {d}t\right) }, \end{aligned}$$

where the continuum partition function $\varvec{Z}^{\alpha ; W}_{T, \beta , h}$ would be defined in analogy to (1.5). The problem is that a.e. realization of the $\alpha$-stable regenerative set $\varvec{\tau }$ has zero Lebesgue measure, hence the integral in (1.6) vanishes, yielding the “trivial” definition $\varvec{\mathrm {P}}^{\alpha ;W}_{T, \beta , h} = \varvec{\mathrm {P}}^\alpha$.

These difficulties turn out to be substantial and not just technical: as we shall see, a non-trivial scaling limit of $\mathrm P^{\omega }_{N,\beta ,h}$ does exist, but, for $\alpha \in \left( \frac{1}{2},1\right)$, it is *not* absolutely continuous with respect to the law $\varvec{\mathrm {P}}^\alpha$ [hence no formula like (1.6) can hold]. Note that an analogous phenomenon happens for the directed polymer in random environment [4].

### Main results

We need to formulate an additional assumption on the renewal processes that we consider. Introducing the renewal function

$$\begin{aligned} u(n) := \mathrm P(n\in \tau ) = \sum _{k=0}^\infty \mathrm P(\tau _k = n) , \end{aligned}$$

assumption (1.1) yields $u(n+\ell ) / u(n) \rightarrow 1$ as $n\rightarrow \infty$, provided $\ell = o(n)$ (see (2.10) below). We ask that the rate of this convergence is at least a power-law of $\frac{\ell }{n}$:

1.7 $$\begin{aligned} \exists C, n_0 \!\in \! (0,\infty ), \ \varepsilon ,\delta \!\in \! (0,1]: \qquad \bigg | \frac{u(n+\ell )}{u(n)} - 1 \bigg | \le C \bigg (\frac{\ell }{n}\bigg )^\delta \quad \ \forall n \ge n_0, \ 0 \le \ell \le \varepsilon n. \end{aligned}$$

#### *Remark 1.2*

As we discuss in Appendix , condition (1.7) is a *very mild* smoothness requirement, that can be verified in most situations. E.g., it was shown by Alexander [2] that for any $\alpha >0$ and slowly varying $L(\cdot )$, there exists a Markov chain $X$ on ${\mathbb {N}}_0$ with $\pm 1$ steps, called Bessel-like random walk, whose return time to $0$, denoted by $T$, is such that

1.8 $$\begin{aligned} K(n) := \mathrm P(T=2n) = \frac{\widetilde{L}(n)}{n^{1+\alpha }} \qquad \text{ as } n\rightarrow \infty , \qquad \text {with}\, \widetilde{L}(n) \sim L(n). \end{aligned}$$

We prove in Appendix  that any such walk always satisfies (1.7).

Recall that ${\mathcal C}$ denotes the compact Polish space of closed subsets of ${\mathbb {R}}$. We denote by ${\mathcal M} _1({\mathcal C} )$ the space of Borel probability measures on ${\mathcal C}$, which is itself a compact Polish space, equipped with the topology of weak convergence. We will work with a *conditioned* version of the disordered pinning model (1.4), defined by

1.9 $$\begin{aligned} \mathrm P^{\omega , \mathrm c}_{N,\beta ,h}(\,\cdot \,) := \mathrm P^{\omega }_{N,\beta ,h}(\,\cdot \,| N \in \tau ). \end{aligned}$$

(In order to lighten notation, when $N \not \in {\mathbb {N}}$ we agree that $\mathrm P^{\omega , \mathrm c}_{N,\beta ,h} := \mathrm P^{\omega , \mathrm c}_{\lfloor N \rfloor ,\beta ,h}$).

Recalling (1.2), let us introduce the notation

1.10 $$\begin{aligned} \mathrm P^{\omega ,\mathrm c}_{NT , \beta _N ,h_N}(\mathrm {d}(\tau /N)) := \text { law of the rescaled set } \frac{\tau }{N} \cap [0,T] \text { under } \mathrm P^{\omega ,\mathrm c}_{NT , \beta _N ,h_N}. \end{aligned}$$

For a fixed realization of the disorder $\omega$, $\mathrm P^{\omega ,\mathrm c}_{NT , \beta _N ,h_N}(\mathrm {d}(\tau /N))$ is a probability law on ${\mathcal C}$, i.e. an element of ${\mathcal M} _1({\mathcal C} )$. Since $\omega$ is chosen randomly, the law $\mathrm P^{\omega ,\mathrm c}_{NT , \beta _N ,h_N}(\mathrm {d}(\tau /N))$ is a *random* element of ${\mathcal M} _1({\mathcal C} )$, i.e. a ${\mathcal M} _1({\mathcal C} )$-valued random variable.

Our first main result is the convergence in distribution of this random variable, provided $\alpha \in \left( \frac{1}{2},1\right)$ and the coupling constants $\beta = \beta _N$ and $h = h_N$ are rescaled appropriately:

1.11 $$\begin{aligned} \beta _N := {\hat{\beta }}\frac{L(N)}{ N^{\alpha - \frac{1}{2}}} \,, \qquad h_N := {\hat{h}}\frac{L(N)}{ N^{\alpha } } \,, \qquad \text {for} \ N\in {\mathbb {N}}, \ {\hat{\beta }}> 0, \ {\hat{h}}\in {\mathbb {R}}. \end{aligned}$$

#### **Theorem 1.3**

(Existence and universality of the CDPM) Fix $\alpha \in (\frac{1}{2},1)$, $T>0$, ${\hat{\beta }}> 0$, ${\hat{h}}\in {\mathbb {R}}$. There exists a ${\mathcal M} _1({\mathcal C} )$-valued random variable $\varvec{\mathrm {P}}^{\alpha ;W,\mathrm c}_{T, {\hat{\beta }}, {\hat{h}}}$, called the (conditioned) *continuum disordered pinning model (CDPM)*, which is a function of the parameters $(\alpha , T, {\hat{\beta }}, {\hat{h}})$ and of a standard Brownian motion $W = (W_t)_{t\ge 0}$, with the following property:

- for any renewal process $\tau$ satisfying (1.1) and (1.7), and $\beta _N$, $h_N$ defined as in (1.11);
- for any i.i.d. sequence $\omega$ satisfying (1.3);

the law $\mathrm P^{\omega ,\mathrm c}_{NT , \beta _N ,h_N}(\mathrm {d}(\tau /N))$ of the rescaled pinning model (1.10), viewed as a ${\mathcal M} _1({\mathcal C} )$-valued random variable, converges in distribution to $\varvec{\mathrm {P}}^{\alpha ;W,\mathrm c}_{T, {\hat{\beta }}, {\hat{h}}}$ as $N\rightarrow \infty$.

We refer to Sect. 1.4 for a discussion on the universality of the CDPM. We stress that the restriction $\alpha \in \left( \frac{1}{2},1\right)$ is substantial and not technical, being linked with the issue of *disorder relevance*, as we explain in Sect. 1.4 (see also [10]).

Let us give a quick explanation of the choice of scalings (1.11). This is the canonical scaling under which the partition function $Z^\omega _{N,\beta _N,h_N}$ in (1.5) has a nontrivial continuum limit. To see this, write

$$\begin{aligned} Z^\omega _{N,\beta ,h}&=\mathrm E\left[ \prod _{n=1}^N\Big (1+\varepsilon ^{\beta ,h}_n\,1\!\!1_{n\in \tau }\Big )\right] \\&=1+\sum _{k=1}^N\sum _{1\le n_1<\cdots <n_k\le N} \varepsilon ^{\beta ,h}_{n_1}\cdots \varepsilon ^{\beta ,h}_{n_k} \,\mathrm P(n_1\in \tau ,...,n_k\in \tau ), \end{aligned}$$

where $\varepsilon ^{\beta ,h}_{n}:=e^{\beta \omega _n-\Lambda (\beta )+h}-1$. By Taylor expansion, as $\beta ,h$ tend to zero, one has the asymptotic behavior ${\mathbb E} [\varepsilon ^{\beta ,h}_{n}] \approx h$ and ${{\mathrm{\mathbb {V}ar}}}(\varepsilon ^{\beta ,h}_{n}) \approx \beta ^2$. Using this fact, we see that the asymptotic mean and variance behavior of the first term ($k=1$) in the above series is

$$\begin{aligned} {\mathbb E} \left[ \sum _{n=1}^N \varepsilon ^{\beta ,h}_{n} \mathrm P(n\in \tau ) \,\right]&\approx h \sum _{n=1}^N \mathrm P(n\in \tau ) \approx h\frac{N^\alpha }{L(N)}, \\ {{\mathrm{\mathbb {V}ar}}}\left[ \sum _{n=1}^N \varepsilon ^{\beta ,h}_{n} \mathrm P(n\in \tau ) \,\right]&\approx \beta ^2 \sum _{n=1}^N \mathrm P(n\in \tau )^2 \approx \beta ^2\frac{N^{2\alpha -1}}{L(N)^2}, \end{aligned}$$

because $\mathrm P(n\in \tau ) \approx n^{\alpha -1} / L(n)$, by (1.1) (see (2.10) below). Therefore, for these quantities to have a non-trivial limit as $N$ tends to infinity, we are forced to scale $\beta _N$ and $h_N$ as in (1.11). Remarkably, this is also the correct scaling for higher order terms in the expansion for $Z^\omega _{N,\beta ,h}$, as well as for the measure $\mathrm P^\omega _{N,\beta _N,h_N}$ to converge to a non-trivial limit.

We now describe the continuum measure. For a fixed realization of the Brownian motion $W = (W_t)_{t\in [0,\infty )}$, which represents the “continuum disorder”, we call $\varvec{\mathrm {P}}^{\alpha ; W,\mathrm c}_{T, {\hat{\beta }}, {\hat{h}}}$ the *quenched law* of the CDPM, while

1.12 $$\begin{aligned} {\mathbb E} \Big [ \varvec{\mathrm {P}}^{\alpha ; W,\mathrm c}_{T, {\hat{\beta }}, {\hat{h}}} \Big ] (\,\cdot \,) := \int \varvec{\mathrm {P}}^{\alpha ; W,\mathrm c}_{T, {\hat{\beta }}, {\hat{h}}} (\,\cdot \,) \, {\mathbb P} (\mathrm {d}W) \end{aligned}$$

will be called the *averaged law* of the CDPM. We also introduce, for $T > 0$, the law $\varvec{\mathrm {P}}_{T}^{\alpha ; \mathrm c}$ of the $\alpha$-stable regenerative set $\varvec{\tau }$ restricted on $[0,T]$ and conditioned to visit $T$:

1.13 $$\begin{aligned} \varvec{\mathrm {P}}_{T}^{\alpha ; \mathrm c}(\,\cdot \,) := \varvec{\mathrm {P}}^\alpha ( \varvec{\tau }\cap [0,T] \in \,\cdot \,|\, T \in \varvec{\tau }), \end{aligned}$$

which will be called the *reference law*. (Relation (1.13) is defined through regular conditional distributions.) Note that both ${\mathbb E} \big [\varvec{\mathrm {P}}^{\alpha ; W,\mathrm c}_{T, {\hat{\beta }}, {\hat{h}}}\big ]$ and $\varvec{\mathrm {P}}_{T}^{\alpha ; \mathrm c}$ are probability laws on ${\mathcal C}$, while $\varvec{\mathrm {P}}^{\alpha ; W,\mathrm c}_{T, {\hat{\beta }}, {\hat{h}}}$ is a *random* probability law on ${\mathcal C}$.

Intuitively, the quenched law $\varvec{\mathrm {P}}^{\alpha ; W,\mathrm c}_{T, {\hat{\beta }}, {\hat{h}}}$ could be conceived as a “Gibbs transformation” of the reference law $\varvec{\mathrm {P}}_{T}^{\alpha ; \mathrm c}$, where each visited site $t \in \varvec{\tau }\cap [0,T]$ of the $\alpha$-stable regenerative set is given a reward/penalty ${\hat{\beta }}\frac{\mathrm {d}W_t}{\mathrm {d}t} + {\hat{h}}$, like in the discrete case. This heuristic interpretation should be taken with care, however, as the following results show.

#### **Theorem 1.4**

(Absolute continuity of the averaged CDPM) For all $\alpha \in \left( \frac{1}{2},1\right)$, $T>0$, ${\hat{\beta }}> 0$, ${\hat{h}}\in {\mathbb {R}}$, the averaged law ${\mathbb E} \big [\varvec{\mathrm {P}}^{\alpha ; W, \mathrm c}_{T, \hat{\beta }, \hat{h}}\big ]$ of the CDPM is absolutely continuous with respect to the reference law $\varvec{\mathrm {P}}^{\alpha ; \mathrm c}_{T}$. It follows that any typical property of the reference law $\varvec{\mathrm {P}}^{\alpha ; \mathrm c}_{T}$ is also a typical property of the quenched law $\varvec{\mathrm {P}}^{\alpha ; W, \mathrm c}_{T, \hat{\beta }, \hat{h}}$, for a.e. realization of $W$:

1.14 $$\begin{aligned} \forall A \subseteq {\mathcal C} \text { such that } \varvec{\mathrm {P}}^{\alpha ; \mathrm c}_{T}(A) = 1: \qquad \varvec{\mathrm {P}}^{\alpha ; W, \mathrm c}_{T, \hat{\beta }, \hat{h}}(A) = 1 \text { for}\, {\mathbb P} \hbox {-a.e. } W. \end{aligned}$$

In particular, for a.e. realization of $W$, the quenched law $\varvec{\mathrm {P}}^{\alpha ; W, \mathrm c}_{T, \hat{\beta }, \hat{h}}$ of the CDPM is supported on closed subsets of $[0,T]$ with Hausdorff dimension $\alpha$.

It is tempting to deduce from (1.14) the absolute continuity of the quenched law $\varvec{\mathrm {P}}^{\alpha ; W, \mathrm c}_{T, \hat{\beta }, \hat{h}}$ with respect to the reference law $\varvec{\mathrm {P}}^{\alpha ; \mathrm c}_{T}$, for a.e. realization of $W$, *but this is false*.

#### **Theorem 1.5**

(Singularity of the quenched CDPM) For all $\alpha \in \left( \frac{1}{2},1\right)$, $T>0$, ${\hat{\beta }}> 0$, ${\hat{h}}\in {\mathbb {R}}$ and for a.e. realization of $W$, the quenched law $\varvec{\mathrm {P}}^{\alpha ; W, \mathrm c}_{T, \hat{\beta }, \hat{h}}$ of the CDPM is singular with respect to the reference law $\varvec{\mathrm {P}}^{\alpha ; \mathrm c}_{T}$:

1.15 $$\begin{aligned} \hbox { for }{\mathbb P} \hbox {-a.e. }W, \exists A \subseteq {\mathcal C} \ \text { such that } \ \varvec{\mathrm {P}}^{\alpha ; \mathrm c}_{T}(A) = 1 \ \text { and } \ \varvec{\mathrm {P}}^{\alpha ; W, \mathrm c}_{T, \hat{\beta }, \hat{h}}(A) = 0. \end{aligned}$$

The seeming contradiction between (1.14) and (1.15) is resolved noting that in (1.14) one cannot exchange “$\forall A \subseteq {\mathcal C}$” and “for ${\mathbb P}$-a.e. $W$”, because there are uncountably many $A \subseteq {\mathcal C}$ (and, of course, the set $A$ appearing in (1.15) depends on the realization of $W$).

We conclude our main results with an explicit characterization of the CDPM. As we discuss in Appendix , each closed subset $C \subseteq {\mathbb {R}}$ can be identified with two non-decreasing and right-continuous functions $\mathtt g_t(C)$ and $\mathtt d_t(C)$, defined for $t\in {\mathbb {R}}$ by

1.16 $$\begin{aligned} \mathtt g_t(C) := \sup \{x: \ x\in C\cap [-\infty , t]\}, \qquad \mathtt d_t(C) := \inf \{x: \ x\in C \cap (t, \infty ]\}. \end{aligned}$$

As a consequence, the law of a *random* closed subset $X \subseteq {\mathbb {R}}$ is uniquely determined by the finite dimensional distributions of the random functions $(\mathtt g_t(X))_{t\in {\mathbb {R}}}$ and $(\mathtt d_t(X))_{t\in {\mathbb {R}}}$, i.e. by the probability laws on $\overline{{\mathbb {R}}}^{2k}$ given, for $k \in {\mathbb {N}}$ and $-\infty < t_1 < t_2 < \ldots < t_k < \infty$, by

1.17 $$\begin{aligned} \mathrm P\big ( \mathtt g_{t_1}(X) \in \mathrm {d}x_1, \, \mathtt d_{t_1}(X) \in \mathrm {d}y_1, \, \ldots , \, \mathtt g_{t_k}(X) \in \mathrm {d}x_k, \, \mathtt d_{t_k}(X) \in \mathrm {d}y_k \big ). \end{aligned}$$

As a further simplification, it is enough to focus on the event that $X \cap [t_i, t_{i+1}] \ne \emptyset$ for all $i=1,\ldots , k$, that is, one can restrict $(x_1, y_1, \ldots , x_k, y_k)$ in (1.17) on the following set:

1.18 $$\begin{aligned}&{\mathcal R} _{t_0, \ldots , t_{k+1}}^{(k)} := \big \{(x_1, y_1, \ldots , x_k, y_k) : \ x_i \in [t_{i-1},t_i], \ y_i \in [t_i,t_{i+1}] \hbox { for } i=1,\ldots , k, \nonumber \\&\qquad \qquad \qquad \qquad \hbox { such that } y_{i} \le x_{i+1} \hbox { for } i=1,\ldots , k-1\big \}, \end{aligned}$$

with $t_0 = -\infty$ and $t_{k+1} := +\infty$. The measures (1.17) restricted on the set (1.18) will be called *restricted finite-dimensional distributions* (f.d.d.) of the random set $X$ (see §).

We can characterize the CDPM by specifying its restricted f.d.d.. We need two ingredients:

1. The restricted f.d.d. of the $\alpha$-stable regenerative set conditioned to visit $T$, i.e. of the reference law $\varvec{\mathrm {P}}_T^{\alpha ;\mathrm c}$ in (1.13): by Proposition A.8, these are absolutely continuous with respect to the Lebesgue measure on ${\mathbb {R}}^{2k}$, with the following density (with $y_{0} := 0$):
  1.19 $$\begin{aligned} \mathsf {f}_{T;t_1,\ldots ,t_k}^{\alpha ;\mathrm c}(x_1,y_1,\ldots ,x_k,y_k)&= \Bigg ( \prod _{i=1}^{k} \frac{C_\alpha }{(x_{i} - y_{i-1})^{1-\alpha } \, (y_i-x_i)^{1+\alpha }} \Bigg ) \frac{T^{1-\alpha }}{(T-y_k)^{1-\alpha }} \,, \end{aligned}$$
  1.20 $$\begin{aligned} \text {with} \quad C_\alpha&:= \,\frac{\alpha \sin (\pi \alpha )}{\pi }, \end{aligned}$$
  where we restrict $(x_1, y_1, \ldots , x_k, y_k)$ on the set (1.18), with $t_0 = 0$ and $t_{k+1} := T$.
2. A family of *continuum partition functions* for our model:
  $$\begin{aligned} \big (\varvec{Z}^{\alpha ; W, \mathrm c}_{\hat{\beta }, \hat{h}}(s, t)\big )_{0\le s\le t <\infty }. \end{aligned}$$
  These were constructed in [10] as the limit, in the sense of finite-dimensional distributions, of the following discrete family (under an appropriate rescaling):
  1.21 $$\begin{aligned} Z_{\beta ,h}^{\omega ,\mathrm c}(a,b):=\mathrm E\Big [ e^{\sum _{n=a+1}^{b-1} (\beta \omega _n -\Lambda (\beta ) + h) 1\!\!1_{\{n\in \tau \}}}\,\Big | a\in \tau , \, b \in \tau \Big ], \qquad 0\le a\le b. \end{aligned}$$
  In Sect. 2 we upgrade the f.d.d. convergence to the process level, deducing important a.s. properties, such as strict positivity and continuity (Theorems 2.1 and 2.4).

We can finally characterize the restricted f.d.d. of the CDPM as follows.

#### **Theorem 1.6**

(F.d.d. of the CDPM) Fix $\alpha \in \left( \frac{1}{2},1\right)$, $T>0$, ${\hat{\beta }}> 0$, ${\hat{h}}\in {\mathbb {R}}$ and let $\big (\varvec{Z}^{\alpha ; W, \mathrm c}_{\hat{\beta }, \hat{h}}(s, t)\big )_{0\le s\le t < \infty }$ be an a.s. continuous version of the continuum partition functions. For a.e. realization of $W$, the quenched law $\varvec{\mathrm {P}}^{\alpha ; W, \mathrm c}_{T, \hat{\beta }, \hat{h}}$ of the CDPM (Theorem 1.3) can be defined as the unique probability law on ${\mathcal C}$ which satisfies the following properties:

(i) $\varvec{\mathrm {P}}^{\alpha ; W, \mathrm c}_{T, \hat{\beta }, \hat{h}}$ is supported on closed subsets $\varvec{\tau }\subseteq [0,T]$ with $\{0, T\} \subseteq \varvec{\tau }$.
(ii) For all $k\in {\mathbb {N}}$ and $0 =: t_0 <t_1<\cdots <t_k < t_{k+1} := T$, and for $(x_1, y_1, \ldots , x_k, y_k)$ restricted on the set ${\mathcal R} ^{(k)}_{t_0,\ldots , t_{k+1}}$ in (1.18), the f.d.d. of $\varvec{\mathrm {P}}^{\alpha ; W, \mathrm c}_{T, \hat{\beta }, \hat{h}}$ have densities given by
  1.22 $$\begin{aligned}&\frac{\varvec{\mathrm {P}}^{\alpha ; W, \mathrm c}_{T, \hat{\beta }, \hat{h}}\big (\mathtt g_{t_1}(\varvec{\tau }) \in \mathrm {d}x_1,\, \mathtt d_{t_1}(\varvec{\tau }) \in \mathrm {d}y_1, \ldots , \mathtt g_{t_k}(\varvec{\tau })\in \mathrm {d}x_k,\, \mathtt d_{t_k}(\varvec{\tau }) \in \mathrm {d}y_k\big )}{\mathrm {d}x_1\, \mathrm {d}y_1 \, \cdots \mathrm {d}x_k\, \mathrm {d}y_k} \nonumber \\&\ \ =\, \left( \, \frac{\prod _{i=0}^k \varvec{Z}^{\alpha ; W, \mathrm c}_{\hat{\beta }, \hat{h}}(y_{i},x_{i+1})}{\varvec{Z}^{\alpha ; W, \mathrm c}_{\hat{\beta }, \hat{h}}(0,T)} \right) \, \mathsf {f}_{T;t_1,\ldots ,t_k}^{\alpha ;\mathrm c}(x_1,y_1,\ldots ,x_k,y_k), \end{aligned}$$
  where we set $y_0 := 0$ and $x_{k+1} := T$, and where $\mathsf {f}_{T;t_1,\ldots ,t_k}^{\alpha ;\mathrm c}(\cdot )$ is defined in (1.19).

### Discussion and perspectives

We conclude the introduction with some observations on the results stated so far, putting them in the context of the existing literature, stating some conjectures and outlining further directions of research.

1. (*Disorder relevance*) The parameter $\beta$ tunes the strength of the disorder in the model $\mathrm P^{\omega , \mathrm c}_{N,\beta ,h}$ (1.9), (1.4). When $\beta =0$, the sequence $\omega$ disappears and we obtain the so-called *homogeneous pinning model*. Roughly speaking, the effect of disorder is said to be:

- *irrelevant* if the disordered model ($\beta > 0$) has the same qualitative behavior as the homogeneous model ($\beta = 0$), provided the disorder is sufficiently weak ($\beta \ll 1$);
- *relevant* if, on the other hand, an arbitrarily small amount of disorder (any $\beta > 0$) alters the qualitative behavior of the homogeneous model ($\beta = 0$).

Recalling that $\alpha$ is the exponent appearing in (1.1), it is known that disorder is irrelevant for pinning models when $\alpha < \frac{1}{2}$ and relevant when $\alpha > \frac{1}{2}$, while the case $\alpha = \frac{1}{2}$ is called marginal and is more delicate (see [20] and the references therein for an overview).

It is natural to interpret our results from this perspective. For simplicity, in the sequel we set $h_N := {\hat{h}}\, L(N)/N^\alpha$, as in (1.11), and we use the notation $\mathrm P^{\omega ,\mathrm c}_{NT , \beta _N ,h_N}(\mathrm {d}(\tau /N))$ (1.10), for the law of the rescaled set $\tau /N$ under the pinning model.

In the homogeneous case ($\beta = 0$), it was shown in [31, Theorem 3.1]^1^ that the weak limit of $\mathrm P^{\alpha ; \mathrm c}_{NT ,0,h_N}(\mathrm {d}(\tau /N))$ as $N\rightarrow \infty$ is a probability law $\varvec{\mathrm {P}}_{T, 0, {\hat{h}}}^{\alpha ; \mathrm c}$ on ${\mathcal C}$ which is *absolutely continuous* with respect to the reference law $\varvec{\mathrm {P}}_{T}^{\alpha ; \mathrm c}$ (recall (1.13)):

1.23 $$\begin{aligned} \frac{\mathrm {d}\varvec{\mathrm {P}}_{T, 0, {\hat{h}}}^{\alpha ; \mathrm c}}{\mathrm {d}\varvec{\mathrm {P}}_{T}^{\alpha ; \mathrm c}}(\varvec{\tau }) = \frac{e^{{\hat{h}}{\mathcal L} _T(\varvec{\tau })}}{\varvec{\mathrm {E}}[e^{{\hat{h}}{\mathcal L} _T(\varvec{\tau })}]} \,, \end{aligned}$$

where ${\mathcal L} _T(\varvec{\tau })$ denotes the so-called local time associated to the regenerative set $\varvec{\tau }$. We stress that this result holds with *no restriction on* $\alpha \in (0,1)$.

Turning to the disordered model $\beta > 0$, what happens for $\alpha \in \left( 0,\frac{1}{2}\right)$? In analogy with [9, 11], we conjecture that *for fixed* $\beta > 0$ *small enough*, the limit in distribution of $\mathrm P_{NT, \beta , h_N}^{\omega , \mathrm c}(\mathrm {d}(\tau /N))$ as $N\rightarrow \infty$ *is the same as for the homogeneous model* ($\beta = 0$), i.e. the law $\varvec{\mathrm {P}}_{T, 0, {\hat{h}}}^{\alpha ; \mathrm c}$ defined in (1.23). Thus, for $\alpha \in \left( 0,\frac{1}{2}\right)$, the continuum model is *non-disordered* (deterministic) and absolutely continuous with respect to the reference law.

This is in striking contrast with the case $\alpha \in \left( \frac{1}{2},1\right)$, where our results show that the continuum model $\varvec{\mathrm {P}}_{T, {\hat{\beta }}, {\hat{h}}}^{\alpha ; W, \mathrm c}$ is truly *disordered* and singular with respect to the reference law (Theorems 1.3, 1.4, 1.5). In other terms, for $\alpha \in \left( \frac{1}{2},1\right)$, *disorder survives in the scaling limit* (even though $\beta _N, h_N \rightarrow 0$) and breaks down the absolute continuity with respect to the reference law, providing a clear manifestation of disorder relevance.

We refer to [10] for a general discussion on disorder relevance in our framework.

2. (*Universality*) The quenched law $\varvec{\mathrm {P}}_{T, {\hat{\beta }}, {\hat{h}}}^{\alpha ; W, \mathrm c}$ of the CDPM is a *random probability law on* ${\mathcal C}$, i.e. a random variable taking values in ${\mathcal M} _1({\mathcal C} )$. Its distribution is a probability law on the space ${\mathcal M} _1({\mathcal C} )$—i.e. an element of ${\mathcal M} _1({\mathcal M} _1({\mathcal C} ))$—which is *universal*: it depends on few macroscopic parameters (the time horizon $T$, the disorder strength and bias ${\hat{\beta }}, {\hat{h}}$ and the exponent $\alpha$) but not on finer details of the discrete model from which it arises, such as the distributions of $\omega _1$ and of $\tau _1$: all these details disappear in the scaling limit.

Another important universal aspect of the CDPM is linked to *phase transitions*. We do not explore this issue here, referring to [10, §1.3] for a detailed discussion, but we mention that the CDPM leads to *sharp predictions* about the asymptotic behavior of the *free energy* and *critical curve* of discrete pinning models, in the weak disorder regime $\lambda , h \rightarrow 0$.

3. (*Bessel processes*) In this paper we consider pinning models built on top of general renewal processeses $\tau = (\tau _k)_{k\in {\mathbb {N}}_0}$ satisfying (1.1) and (1.7). In the special case when the renewal process is the zero level set of a Bessel-like random walk [1] (recall Remark 1.2), one can define the pinning model (1.4), (1.9) as a probability law on random walk paths (and not only on their zero level set).

Rescaling the paths diffusively, one has an analogue of Theorem 1.3, in which the CDPM is built as a random probability law on the space $C([0,T], {\mathbb {R}})$ of continuous functions from $[0,T]$ to ${\mathbb {R}}$. Such an extended CDPM is a continuous process $(\varvec{X}_t)_{t\in [0,T]}$, that can be heuristically described as a *Bessel process of dimension* $\delta =2(1-\alpha )$ *interacting with an independent Brownian environment* $W$ *each time* $X_t = 0$. The “original” CDPM of our Theorem 1.3 corresponds to the zero level set $\varvec{\tau }:= \{t\in [0,T]: \varvec{X}_t = 0\}$.

We stress that, starting from the zero level set $\varvec{\tau }$, one can reconstruct the whole process $(\varvec{X}_t)_{t\in [0,T]}$ by pasting independent Bessel excusions on top of $\varvec{\tau }$ (more precisely, since the open set $[0,T] {\setminus } \varvec{\tau }$ is a countable union of disjoint open intervals, one attaches a Bessel excursion to each of these intervals).^2^ This provides a rigorous definition of $(\varvec{X}_t)_{t\in [0,T]}$ in terms of $\varvec{\tau }$ and shows that the zero level set is indeed the fundamental object.

4. (*Infinite-volume limit*) Our continuum model $\varvec{\mathrm {P}}_{T, {\hat{\beta }}, {\hat{h}}}^{\alpha ; W, \mathrm c}$ is built on a finite interval $[0,T]$. An interesting open problem is to let $T \rightarrow \infty$, proving that $\varvec{\mathrm {P}}_{T, {\hat{\beta }}, {\hat{h}}}^{\alpha ; W, \mathrm c}$ converges in distribution to an *infinite-volume CDPM* $\varvec{\mathrm {P}}_{\infty , {\hat{\beta }}, {\hat{h}}}^{\alpha ; W, \mathrm c}$. Such a limit law would inherit scaling properties from the continuum partition functions, Theorem 2.4 (iii). (See also [29] for related work in the non-disordered case ${\hat{\beta }}= 0$).

### Organization of the paper

The rest of the paper is organized as follows.

- In Sect. 2, we study the properties of continuum partition functions.
- In Sect. 3, we prove Theorem 1.6 on the characterization of the CDPM, which also yields Theorem 1.3.
- In Sect. 4, we prove Theorems 1.4 and 1.5 on the relations between the CDPM and the $\alpha$-stable regenerative set.
- In Appendix , we describe the measure-theoretic background needed to study random closed subsets of ${\mathbb {R}}$, which is of independent interest.
- Lastly, in Appendices  and C we prove some auxiliary estimates.

## Continuum partition functions as a process

In this section we focus on a family $\big (\varvec{Z}^{\alpha ; W, \mathrm c}_{\hat{\beta }, \hat{h}}(s, t)\big )_{0\le s\le t <\infty }$ of *continuum partition functions* for our model, which was recently introduced in [10] as the limit of the discrete family (1.21) in the sense of finite-dimensional distributions. We upgrade this convergence to the process level (Theorem 2.1), which allows us to deduce important properties (Theorem 2.4). Besides their own interest, these results are the key to the construction of the CDPM.

### Fine properties of continuum partition functions

Recalling (1.21), where $Z_{\beta ,h}^{\omega , \mathrm c}(a, b)$ is defined for $a,b\in {\mathbb {N}}_0$, we extend $Z_{\beta ,h}^{\omega , \mathrm c}(\cdot , \cdot )$ to a continuous function on

$$\begin{aligned}{}[0,\infty )^2_\le :=\{(s,t) \in [0,\infty )^2: \ 0\le s\le t <\infty \}, \end{aligned}$$

bisecting each unit square $[m-1,m]\times [n-1,n]$, with $m\le n\in {\mathbb {N}}$, along the main diagonal and linearly interpolating $Z_{\beta ,h}^{\omega , \mathrm c}(\cdot , \cdot )$ on each triangle. In this way, we can regard

2.1 $$\begin{aligned} \big (Z^{\omega ,\mathrm c}_{\beta _N,h_N}(sN, tN)\big )_{0\le s\le t <\infty } \end{aligned}$$

as random variables taking values in the space $C([0,\infty )^2_\le , {\mathbb {R}})$, equipped with the topology of uniform convergence on compact sets and with the corresponding Borel $\sigma$-algebra. The randomness comes from the disorder sequence $\omega = (\omega _n)_{n\in {\mathbb {N}}}$.

Even though our main interest in this paper is for $\alpha \in \left( \frac{1}{2},1\right)$, we also include the case $\alpha > 1$ in the following key result, which is proved in Sect. 2.2 below.

#### **Theorem 2.1**

(Process level convergence of partition functions) Let $\alpha \in \left( \frac{1}{2}, 1\right) \cup (1,\infty )$, $\hat{\beta }> 0$, $\hat{h}\in {\mathbb {R}}$. Let $\tau$ be a renewal process satisfying (1.1) and (1.7), and $\omega$ be an i.i.d. sequence satisfying (1.3). For every $N\in {\mathbb {N}}$, define $\beta _N, h_N$ by (recall (1.11))

2.2 $$\begin{aligned} {\left\{ \begin{array}{ll} \displaystyle \beta _N := {\hat{\beta }}\frac{L(N)}{ N^{\alpha - \frac{1}{2}}} \\ \displaystyle h_N := {\hat{h}}\frac{L(N)}{ N^{\alpha } } \end{array}\right. } \ \text {for } \alpha \in \left( \tfrac{1}{2},1\right) , \qquad {\left\{ \begin{array}{ll} \displaystyle \beta _N := \frac{{\hat{\beta }}}{\sqrt{N}} \\ \displaystyle h_N := \frac{{\hat{h}}}{N} \end{array}\right. } \ \text {for } \alpha > 1. \end{aligned}$$

As $N\rightarrow \infty$ the two-parameter family $\big (Z^{\omega ,\mathrm c}_{\beta _N,h_N}(sN, tN) \big )_{0\le s\le t <\infty }$ converges in distribution on $C([0,\infty )^2_\le , {\mathbb {R}})$ to a family $\big (\varvec{Z}^{\alpha ; W, \mathrm c}_{\hat{\beta }, \hat{h}}(s, t)\big )_{0\le s\le t <\infty }$, called *continuum partition functions*. For all $0\le s\le t<\infty$, one has the Wiener chaos representation

2.3 $$\begin{aligned} \varvec{Z}^{\alpha ; W, \mathrm c}_{\hat{\beta }, \hat{h}}(s, t) = 1 + \sum _{k=1}^\infty \ \ \idotsint \limits _{s<t_1<\cdots <t_k<t} \varvec{\psi }^{\alpha ; \mathrm c}_{s,t}(t_1,\ldots , t_k) \prod _{i=1}^k (\hat{\beta }\, \mathrm {d}W_{t_i} + \hat{h} \, \mathrm {d}t_i), \end{aligned}$$

where $W = (W_t)_{t\ge 0}$ is a standard Brownian motion, the series in (2.3) converges in $L^2$, and the kernel $\varvec{\psi }^{\alpha ; \mathrm c}_{s,t}(t_1,\ldots , t_k)$ is defined as follows, with $C_\alpha$ as in (1.20) and $t_0 := s$:2.4


#### *Remark 2.2*

The integral in (2.3) is defined by expanding formally the product of differentials and reducing to standard multiple Wiener and Lebesgue integrals. An alternative equivalent definition is to note that, by Girsanov’s theorem, the law of $({\hat{\beta }}W_t +{\hat{h}}t)_{t\in [0,T]}$ is absolutely continuous w.r.t. that of $({\hat{\beta }}W_t)_{t\in [0,T]}$, with Radon–Nikodym density

2.5 $$\begin{aligned} \mathfrak {f}_{T, {\hat{\beta }}, {\hat{h}}}(W) := e^{\left( \frac{\hat{h}}{\hat{\beta }}\right) \, W_T - \frac{1}{2} \left( \frac{\hat{h}}{\hat{\beta }}\right) ^2 T} \,. \end{aligned}$$

It follows that $\big (\varvec{Z}^{\alpha ; W, \mathrm c}_{\hat{\beta }, \hat{h}}(s, t)\big )_{0\le s\le t\le T}$ has the same law as $\big (\varvec{Z}^{\alpha ; W, \mathrm c}_{\hat{\beta }, 0}(s, t)\big )_{0\le s\le t\le T}$ (for ${\hat{h}}= 0$) under a change of measure with density (2.5). For further details, see [10].

#### *Remark 2.3*

Theorem 2.1 still holds if we also include the two-parameter family of *unconditioned* partition functions $\big (Z^{\omega }_{\beta _N,h_N}(sN, tN)\big )_{0\le s\le t<\infty }$, defined the same way as $Z^{\omega , \mathrm c}_{\beta , h}(a,b)$ in (1.21), except for removing the conditioning on $b\in \tau$. The limiting process $\varvec{Z}^{\alpha ; W}_{\hat{\beta }, \hat{h}}(s, t)$ will then have a kernel $\varvec{\psi }_{s,t}^{\alpha }$, which modifies $\varvec{\psi }^{\alpha ; \mathrm c}_{s,t}$ in (2.4), by setting

2.6 $$\begin{aligned} \varvec{\psi }^{\alpha }_{s,t}(t_1,\ldots , t_k) = \prod _{i=1}^k \frac{C_\alpha }{(t_i - t_{i-1})^{1-\alpha }}, \qquad \text {if } \alpha \in \left( \tfrac{1}{2}, 1\right) \!. \end{aligned}$$

By Theorem 2.1, we can fix a version of the continuum partition functions $\varvec{Z}^{\alpha ; W, \mathrm c}_{\hat{\beta }, \hat{h}}(s, t)$ which is continuous in $(s,t)$. This will be implicitly done henceforth. We can then state some fundamental properties, proved in Sect. 2.3.

#### **Theorem 2.4**

(Properties of continuum partition functions) For all $\alpha \in \left( \tfrac{1}{2}, 1\right)$, $\hat{\beta }>0$, $\hat{h}\in {\mathbb {R}}$ the following properties hold:

(i) *(Positivity)* For a.e. realization of $W$, the function $(s,t) \mapsto \varvec{Z}^{\alpha ; W, \mathrm c}_{\hat{\beta }, \hat{h}}(s,t)$ is continuous and strictly positive at all $0\le s\le t < \infty$.
(ii) *(Translation Invariance)* For any fixed $t>0$, the process $\left( \varvec{Z}^{\alpha ; W, \mathrm c}_{\hat{\beta }, \hat{h}}(t,t+u)\right) _{u\ge 0}$ has the same distribution as $\left( \varvec{Z}^{\alpha ; W, \mathrm c}_{\hat{\beta }, \hat{h}}(0,u)\right) _{u\ge 0}$, and is independent of $\left( \varvec{Z}^{\alpha ; W, \mathrm c}_{\hat{\beta }, \hat{h}}(s,u)\right) _{0\le s\le u\le t}$.
(iii) *(Scaling Property)* For any constant $A>0$, one has the equality in distribution
  2.7 $$\begin{aligned} \big (\varvec{Z}^{\alpha ; W, \mathrm c}_{\hat{\beta }, \hat{h}}(As, At)\big )_{0\le s\le t <\infty } \mathop {=}\limits ^\mathrm{dist} \Big (\varvec{Z}^{\alpha ; W, \mathrm c}_{A^{\alpha -1/2}\hat{\beta }, A^\alpha \hat{h}}(s, t) \Big )_{0\le s\le t < \infty }. \end{aligned}$$
(iv) *(Renewal Property)* Setting $\varvec{Z}(s,t) := \varvec{Z}^{\alpha ; W, c}_{\hat{\beta }, \hat{h}}(s,t)$ for simplicity, for a.e. realization of $W$ one has, for all $0\le s<u<t < \infty$,
  2.8 $$\begin{aligned} \frac{C_\alpha \,\varvec{Z}(s,t)}{(t-s)^{1-\alpha }} = \int _{x\in (s,u)} \int _{y\in (u,t)} \frac{C_\alpha \,\varvec{Z}(s,x)}{(x-s)^{1-\alpha }} \, \frac{1}{(y-x)^{1+\alpha }} \, \frac{C_\alpha \, \varvec{Z}(y,t)}{(t-y)^{1-\alpha }}\, \mathrm {d}x \, \mathrm {d}y, \end{aligned}$$
  which can be rewritten, recalling (1.19), as follows:
  2.9 $$\begin{aligned} \varvec{Z}(s,t) = \varvec{\mathrm {E}}^{\alpha ; c}_{t-s} \Big [ \varvec{Z}\big ( s,\mathtt g_u(\varvec{\tau }) \big ) \, \varvec{Z}\big ( \mathtt d_u(\varvec{\tau }),t \big ) \Big ]. \end{aligned}$$

The rest of this section is devoted to the proof of Theorems 2.1 and 2.4. We recall that assumption (1.1) entails the following key renewal estimates, with $C_\alpha$ as in (1.20):

2.10 $$\begin{aligned} u(n) \,:=\, \mathrm P(n \in \tau ) \,\sim \, {\left\{ \begin{array}{ll} \displaystyle \frac{C_\alpha }{L(n) n^{1-\alpha }} &{} \text { if } 0 < \alpha < 1, \\ \displaystyle \frac{1}{\mathrm E[\tau _1]} = (const.) \in (0,\infty ) &{} \text { if } \alpha > 1, \end{array}\right. } \end{aligned}$$

by the classical renewal theorem for $\alpha > 1$ and by [12, 18] for $\alpha \in (0,1)$. Let us also note that the additional assumption (1.7) for $\alpha \in (0,1)$ can be rephrased as follows:

2.11 $$\begin{aligned} |u(q) - u(r)| \le C \Big (\frac{r-q}{r}\Big )^\delta u(q), \qquad \forall r \ge q \ge n_0 \ \text { with } \ r-q \le \varepsilon r, \end{aligned}$$

up to a possible change of the constants $C, n_0, \varepsilon$.

### Proof of Theorem 2.1

We may assume $T=1$. For convergence in distribution on $C([0,1]^2_\le ,{\mathbb {R}})$ it suffices to show that $\{(Z^{\omega ,\mathrm c}_{\beta _N,h_N}(sN, tN))_{0\le s\le t\le 1}\}_{N\in {\mathbb {N}}}$ is a tight family, because the finite-dimensional distribution convergence was already obtained in [10] (see Theorem 3.1 and Remark 3.3 therein). We break down the proof into five steps.

Step 1. *Moment criterion*. We recall a moment criterion for the Hölder continuity of a family of multi-dimensional stochastic processes, which was also used in [3] to prove similar tightness results for the directed polymer model. Using Garsia’s inequality [17, Lemma 2] with $\Psi (x)=|x|^p$ and $\varphi (u)= u^q$ for $p\ge 1$ and $pq>2d$, the modulus of continuity of a continuous function $f:[0,1]^d \rightarrow {\mathbb {R}}$ can be controlled by

$$\begin{aligned} |f(x)-f(y)| \le 8 \int _0^{|x-y|} \Psi ^{-1}\Big (\frac{B}{u^{2d}}\Big ) \mathrm {d}\varphi (u)&= 8 \int _0^{|x-y|} \frac{B^{1/p}}{u^{2d/p}} \mathrm {d}(u^q)\\&= \frac{8 B^{1/p}q}{q-2d/p} |x-y|^{q -2d/p}, \end{aligned}$$

where

$$\begin{aligned} B=B(f)&= \iint _{[0,1]^d\times [0,1]^d} \Psi \left( \frac{f(x)-f(y)}{\varphi \left( \frac{x-y}{\sqrt{d}}\right) } \right) \mathrm {d}x\, \mathrm {d}y\\&= d^{q/2} \iint _{[0,1]^d\times [0,1]^d} \frac{|f(x)-f(y)|^p}{|x-y|^{pq}} \mathrm {d}x\, \mathrm {d}y. \end{aligned}$$

Suppose now that $(f_N)_{N\in {\mathbb {N}}}$ are *random* continuous function on $[0,1]^d$ such that

$$\begin{aligned} \mathrm E[|f_N(x)-f_N(y)|^p] \le C|x-y|^\eta , \end{aligned}$$

for some $C, p, q, \eta \in (0,\infty )$ with $pq > 2d$ and $\eta > pq-d$, uniformly in $N\in {\mathbb {N}}$, $x,y \in [0,1]^d$. Then $\mathrm E[B(f_N)]$ is bounded uniformly in $N$, hence $\{B(f_N)\}_{N\in {\mathbb {N}}}$ is tight. If the functions $f_N$ are equibounded at some point (e.g. $f_N(0) = 1$ for every $N\in {\mathbb {N}}$), the tightness of $B(f_N)$ entails the tightness of $\{f_N\}_{N\in {\mathbb {N}}}$, by the Arzelà-Ascoli theorem [6, Theorem 7.3].

To prove the tightness of $\{(Z^{\omega ,\mathrm c}_{\beta _N,h_N}(sN, tN))_{0\le s\le t\le 1}\}_{N\in {\mathbb {N}}}$, it then suffices to show that

2.12 $$\begin{aligned} {\mathbb E} \Big [\big |Z^{\omega , \mathrm c}_{\beta _N, h_N}(s_1N, t_1N) - Z^{\omega , \mathrm c}_{\beta _N, h_N} (s_2N, t_2N)\big |^p \Big ] \le C \big (\sqrt{(s_1-s_2)^2+(t_1-t_2)^2}\,\big )^\eta , \end{aligned}$$

which by triangle inequality, translation invariance and symmetry can be reduced to

2.13 $$\begin{aligned} \exists C > 0, \ p \ge 1, \ \eta > 2: \qquad {\mathbb E} \Big [\big |Z^{\omega , \mathrm c}_{\beta _N, h_N}(0, tN) - Z^{\omega , \mathrm c}_{\beta _N, h_N}(0, sN) \big |^p \Big ] \le C |t-s|^\eta , \end{aligned}$$

uniformly in $N\in {\mathbb {N}}$ and $0\le s<t\le 1$. (Conditions $pq>2d$ and $\eta > pq-d$ are then fulfilled by any $q \in (\frac{4}{p}, \frac{2+\eta }{p})$, since $d=2$). Since $Z^{\omega , \mathrm c}_{\beta _N, h_N}(0, \cdot )$ is defined on $[0,\infty )$ via linear interpolation, it suffices to prove (2.13) for $s,t$ with $sN, tN\in \{0\}\cup {\mathbb {N}}$.

Step 2. *Polynomial chaos expansion* To simplify notation, let us denote

$$\begin{aligned} \Psi _{N,r} := Z^{\omega , \mathrm c}_{\beta _N, h_N}(0, r) = \mathrm E\left[ \prod _{n=1}^{r-1} e^{(\beta _N \omega _n - \Lambda (\beta _N) + h_N) 1\!\!1_{\{n\in \tau \}}} \, \Bigg |\, r\in \tau \right] \qquad \text{ for } r\in {\mathbb {N}}, \end{aligned}$$

and $\Psi _{N,0}:=1$. Since $e^{x 1\!\!1_{\{n\in \tau \}}} = 1 + (e^x-1) 1\!\!1_{\{n\in \tau \}}$ for all $x\in {\mathbb {R}}$, we set

2.14 $$\begin{aligned} \xi _{N,i}:= e^{\beta _N\omega _i -\Lambda (\beta _N) +h_N}-1, \end{aligned}$$

and rewrite $\Psi _{N,r}$ as a *polynomial chaos expansion*:

2.15 $$\begin{aligned} \Psi _{N,r} = \mathrm E\left[ \prod _{i=1}^{r-1} \left( 1+\xi _{N,i} 1\!\!1_{\{i\in \tau \}}\right) \,\Bigg |\, r\in \tau \right] = \sum _{I\subset \{1,\ldots , r-1\}} \mathrm P(I\subset \tau | r\in \tau ) \prod _{i\in I}\xi _{N,i}, \end{aligned}$$

using the notation $\{I\subset \tau \} := \bigcap _{i\in I} \{i\in \tau \}$.

Recalling (2.2) and (1.3), it is easy to check that

2.16 $$\begin{aligned} {\mathbb E} [\xi _{N,i}]&= e^{h_N}-1 = h_N + O(h_N^2), \nonumber \\ \sqrt{{{\mathrm{\mathbb {V}ar}}}(\xi _{N,i})}&= \sqrt{e^{2h_N}\big (e^{\Lambda (2\beta _N)-2\Lambda (\beta _N)}-1\big )} = \sqrt{\beta _N^2 +O(\beta _N^3)} = \beta _N+ O(\beta _N^2),\nonumber \\ \end{aligned}$$

where we used the fact that $h_N = o(\beta _N)$ and we Taylor expanded $\Lambda (t) := \log {\mathbb E} [e^{t\omega _1}]$, noting that $\Lambda (0)= \Lambda '(0)= 0$ and $\Lambda ''(0)= 1$. Thus $h_N$ and $\beta _N$ are approximately the mean and standard deviation of $\xi _{N,i}$. Let us rewrite $\Psi _{N,r}$ in (2.15) using normalized variables $\zeta _{N,i}$:

2.17 $$\begin{aligned} \Psi _{N,r} = \sum _{I\subset \{1,\ldots , r-1\}} \psi _{N,r}(I) \prod _{i\in I}\zeta _{N,i}, \qquad \text {where} \qquad \zeta _{N,i} := \frac{1}{\beta _N} \xi _{N,i}, \end{aligned}$$

where $\psi _{N,r}(\emptyset ):=1$ and for $I=\{n_1 <n_2<\cdots <n_k\}\subset {\mathbb {N}}$, recalling (2.10), we can write

2.18 $$\begin{aligned} \psi _{N,r}(I) = \psi _{N,r}(n_1,\ldots , n_k) := \beta _N^{|I|} \mathrm P(I\subset \tau | r\in \tau ) = (\beta _N)^k \frac{1}{u(r)} \prod _{i=1}^{k+1} u(n_i - n_{i-1}), \end{aligned}$$

with $$n_0 := 0$$, $$n_{k+1} := r$$.

To prove (2.13), we write $Z^{\omega , \mathrm c}_{\beta _N, h_N}(0, sN)=\Psi _{N,q}$ and $Z^{\omega , \mathrm c}_{\beta _N, h_N}(0, tN)=\Psi _{N,r}$, with $q := sN$ and $r := tN$, so that $0\le q<r\le N$. For a given *truncation level* $m = m(q,r,N) \in (0, q)$, that we will later choose as

2.19 $$\begin{aligned} m = m(q,r,N) := {\left\{ \begin{array}{ll} 0 &{} \text {if } q\le \sqrt{N(r-q)} \\ q-\sqrt{N(r-q)} &{} \text {otherwise,} \end{array}\right. } \end{aligned}$$

so that $0\le m<q<r\le N$, we write

$$\begin{aligned} \Psi _{N,r} - \Psi _{N,q} = \Xi _1 +\Xi _2 -\Xi _3 \end{aligned}$$

with

2.20 $$\begin{aligned} \Xi _1 \,&= \, \sum _{I\subset \{1,\ldots , m\}}\big (\psi _{N,r}(I)-\psi _{N,q}(I)\big ) \prod _{i\in I} \zeta _{N,i}, \nonumber \\ \Xi _2 \,&= \, \mathop {\mathop {\sum }\limits _{I\subset \{1,\ldots , r-1\}}}\limits _{I\cap \{m+1, \ldots , r-1\}\ne \emptyset } \psi _{N,r}(I)\prod _{i\in I}\zeta _{N,i}, \qquad \text{ and } \nonumber \\ \Xi _3 \,&= \, \mathop {\mathop {\sum }\limits _{I\subset \{1,\ldots , q-1\}}}\limits _{I\cap \{m+1, \ldots , q-1\}\ne \emptyset } \psi _{N,q}(I)\prod _{i\in I} \zeta _{N,i}. \end{aligned}$$

To establish (2.13) and hence tightness, it suffices to show that for each $i=1,2,3$,

2.21 $$\begin{aligned} \exists C > 0, \ p \ge 1, \ \eta > 2: \quad {\mathbb E} [|\Xi _i|^p] \le C \Big (\frac{r-q}{N}\Big )^\eta \quad \forall N\in {\mathbb {N}}, \ 0\le q<r\le N.\nonumber \\ \end{aligned}$$

Step 3. *Change of measure* We now estimate the moments of $\xi _{N,i}$ defined in (2.14). Since $(a+b)^{2k} \le 2^{2k-1}(a^{2k} + b^{2k})$, for all $k\in {\mathbb {N}}$, and $h_N = O(\beta _N^2)$ by (2.2), we can write

2.22 $$\begin{aligned} {\mathbb E} [\xi _{N,i}^{2k}]&\le 2^{2k-1}e^{2k(h_N-\Lambda (\beta _N))} {\mathbb E} \big [\big (e^{\beta _N\omega _i}-1\big )^{2k}\big ] + 2^{2k-1}(e^{-\Lambda (\beta _N)+h_N}-1)^{2k} \nonumber \\&\le C(k) \, \beta _N^{2k} \, {\mathbb E} \left[ \left( \frac{1}{\beta _N} \int _0^{\beta _N} \omega _i e^{t\omega _i}\mathrm {d}t\right) ^{2k}\right] + O(\beta _N^{4k}+h_N^{2k}) \nonumber \\&\le C(k)\beta _N^{2k-1} \int _0^{\beta _N} {\mathbb E} [\omega _i^{2k}e^{2k t\omega _i}]\mathrm {d}t + o(\beta _N^{2k})\, =\, O(\beta _N^{2k}), \end{aligned}$$

because ${\mathbb E} [\omega _i^{2k} e^{2kt\omega _i}]$ is uniformly bounded for $t\in [0, t_0/4k]$ by our assumption (1.3).

Recalling (2.16), (2.17) and (2.2), the random variables $(\zeta _{N,i})_{i\in {\mathbb {N}}}$ are i.i.d. with

2.23 $$\begin{aligned} {\mathbb E} [\zeta _{N,i}] \,\underset{N\rightarrow \infty }{\sim }\, \frac{{\hat{h}}}{{\hat{\beta }}} \frac{1}{\sqrt{N}} \,, \qquad {{\mathrm{\mathbb {V}ar}}}[\zeta _{N,i}] \,\underset{N\rightarrow \infty }{\sim }\, 1, \qquad \sup _{N,i\in {\mathbb {N}}}{\mathbb E} [(\zeta _{N,i})^{2k}] < \infty . \end{aligned}$$

It follows, in particular, that $\{\zeta _{N,i}^2\}_{i,N\in {\mathbb {N}}}$ are uniformly integrable. We can then apply a change of measure result established in [10, Lemma B.1], which asserts that we can construct i.i.d. random variables $(\widetilde{\zeta }_{N,i})_{i\in {\mathbb {N}}}$ with marginal distribution ${\mathbb P} (\widetilde{\zeta }_{N,i}\in \mathrm {d}x) = f_N(x) {\mathbb P} (\zeta _{N,i}\in \mathrm {d}x)$, for which there exists $C > 0$ such that for all $p\in {\mathbb {R}}$ and $i, N\in {\mathbb {N}}$

2.24 $$\begin{aligned} {\mathbb E} [\widetilde{\zeta }_{N,i}]=0, \qquad {\mathbb E} [\widetilde{\zeta }_{N,i}^2] \le 1 + C/\sqrt{N}, \qquad \text {and} \qquad {\mathbb E} [f_N(\zeta _{N,i})^p] \le 1+ C/N. \end{aligned}$$

Let $\widetilde{\Xi }_i$ be the analogue of $\Xi _i$ constructed from the $\widetilde{\zeta }_{N,i}$’s instead of the $\zeta _{N,i}$’s. By Hölder,

$$\begin{aligned} {\mathbb E} \big [|\Xi _i|^{l-1}\big ]&= {\mathbb E} \left[ |\Xi _i|^{l-1} \prod _{i=1}^N f_N(\zeta _{N,i})^{\frac{l-1}{l}} \prod _{i=1}^N f_N(\zeta _{N,i})^{-\frac{l-1}{l}}\right] \\&\le {\mathbb E} \big [|\widetilde{\Xi }_i|^{l}\big ]^{\frac{l-1}{l}} {\mathbb E} \big [f_N(\zeta _{N,1})^{1-l}\big ]^{\frac{N}{l}} \le e^{\frac{C}{l}} {\mathbb E} \big [|\widetilde{\Xi }_i|^{l}\big ]^{\frac{l-1}{l}}. \end{aligned}$$

Relation (2.21), and hence the tightness of $\{Z^{\omega , \mathrm c}_{\beta _N, h_N}(\cdot , \cdot )\}_{N\in {\mathbb {N}}}$, is thus reduced to showing

2.25 $$\begin{aligned} {\mathbb E} \big [|\widetilde{\Xi }_i|^{l}\big ] \le C \Big (\frac{r-q}{N}\Big )^\eta \qquad \text { for all } N\in {\mathbb {N}}\text { and } 0\le q<r\le N, \end{aligned}$$

for some $l\in {\mathbb {N}}$, $l\ge 2$ and $\eta >0$ satisfying $\eta > 2 \frac{l}{l-1}$.

Step 4. *Bounding* ${\mathbb E} [|\widetilde{\Xi }_2|^{l}]$. We note that the bound for ${\mathbb E} [|\widetilde{\Xi }_3|^{l}]$ is exactly the same as that for ${\mathbb E} [|\widetilde{\Xi }_2|^{l}]$, and hence will be omitted. First we write $\widetilde{\Xi }_2$ as

2.26 $$\begin{aligned} \widetilde{\Xi }_2 = \sum _{k=1}^{r-1} \widetilde{\Xi }_2^{(k)} , \qquad \text {where} \qquad \widetilde{\Xi }_2^{(k)} := \mathop {\mathop {\sum }\limits _{|I|=k, I\subset \{1,\ldots , r-1\}}}\limits _{I\cap \{m+1, \ldots , r-1\}\ne \emptyset } \psi _{N,r}(I)\prod _{i\in I} \widetilde{\zeta }_{N,i}, \end{aligned}$$

with $\widetilde{\Xi }_2^{(k)}$ consisting of all terms of degree $k$. The hypercontractivity established in [26], Prop. 3.16 & 3.12] allows to estimates moments of order $l$ in terms of moments of order $2$: more precisely, setting $\Vert X\Vert _p := \mathrm E[|X|^p]^{1/p}$, we have for all $l\ge 2$

2.27 $$\begin{aligned} \Vert \widetilde{\Xi }_2\Vert _l^l := {\mathbb E} [|\widetilde{\Xi }_2|^{l}] \le \left( \sum _{k=1}^{r-1} \Vert \widetilde{\Xi }_2^{(k)}\Vert _l\right) ^l \le \left( \sum _{k=1}^{r-1} (c_l)^k \Vert \widetilde{\Xi }_2^{(k)}\Vert _2\right) ^l, \end{aligned}$$

where $c_l := 2\sqrt{l-1} \max _{N\in {\mathbb {N}}}\Big ( \frac{\Vert \widetilde{\zeta }_{N,1}\Vert _l}{\Vert \widetilde{\zeta }_{N,1}\Vert _2}\Big )$ is finite and depends only on $l$, by (2.23).

We now turn to the estimation of $\Vert \widetilde{\Xi }_2^{(k)}\Vert _2$. Let us recall the definition of $\psi _{N,r}$ in (2.18). It follows by (2.24) that ${{\mathrm{\mathbb {V}ar}}}(\widetilde{\zeta }_{N,1})\le 1+C/\sqrt{N}\le 2$ for all $N$ large. We then have

2.28 $$\begin{aligned}&\Vert \widetilde{\Xi }_2^{(k)}\Vert _2^2 = {\mathbb E} \big [\big (\widetilde{\Xi }_2^{(k)}\big )^2\big ] = \sum _{y=0}^{k-1} \mathop {\mathop {\sum }\limits _{1\le n_1<\cdots < n_y\le m}}\limits _{m+1\le n_{y+1}<\cdots <n_k\le r-1} \psi ^2_{N,r}(n_1,\ldots , n_k) {{\mathrm{\mathbb {V}ar}}}(\widetilde{\zeta }_{N,1})^k \nonumber \\&\quad \le 2^k \sum _{y=0}^{k-1} \mathop {\mathop {\sum }\limits _{1\le n_1<\cdots <n_y\le m}}\limits _{m+1\le n_{y+1}<\cdots <n_k\le r-1} \frac{\beta _N^{2k}\, u(n_1)^2 u(n_2-n_1)^2\cdots u(r-n_k)^2}{u(r)^2} \nonumber \\&\quad \le 4^k \sum _{y=0}^{k-1} \mathop {\mathop {\!\idotsint }\limits _{0<t_1<\cdots <t_y<\frac{m}{N}}}\limits _ {\frac{m}{N}<t_{y+1}<\cdots <t_{k}<\frac{r}{N}} \!\! \frac{(\sqrt{N}\beta _N u(\lceil N t_1\rceil ))^2 \cdots (\sqrt{N}\beta _N u(r-\lceil N t_k\rceil ))^2}{(\sqrt{N}\beta _N u(r))^2}\ \mathrm {d}t_1\cdots \mathrm {d}t_{k}.\nonumber \\ \end{aligned}$$

It remains to estimate this integral, when $\frac{1}{2}<\alpha <1$ (the case $\alpha > 1$ is easy). By (2.10)

$$\begin{aligned} \frac{1}{c} \frac{1}{L(\ell +1) (\ell +1)^{1-\alpha }} \le u(\ell ) \le c \, \frac{1}{L(\ell +1) (\ell +1)^{1-\alpha }} \qquad \forall \ell \in {\mathbb {N}}\,, \end{aligned}$$

for some $c \in (0,\infty )$. Since $\lceil Nt\rceil -\lceil Ns\rceil + 1 \ge N(t-s)$, recalling (2.2) we obtain

$$\begin{aligned} \sqrt{N}\beta _N u\big (\lceil Nt\rceil -\lceil Ns\rceil \big ) \le c \frac{L(N)}{L\big (\lceil Nt\rceil -\lceil Ns\rceil +1\big )} \frac{1}{(t - s)^{1-\alpha }} \,. \end{aligned}$$

Let us now fix

2.29 $$\begin{aligned} \alpha ':= {\left\{ \begin{array}{ll} 1 &{} \text {when } \alpha >1 \\ \text {any number in } \big (\frac{1}{2}, \alpha \big ) &{} \text {when } \frac{1}{2}<\alpha <1. \end{array}\right. } \end{aligned}$$

Since $L(\cdot )$ is slowly varying, by Potter bounds [8, Theorem 1.5.6] for every $\varepsilon > 0$ there is $D_\varepsilon \in (0,\infty )$ such that $L(a)/L(b) \le D_\varepsilon \max \{ (a/b)^{\varepsilon }, (b/a)^{\varepsilon }\}$ for all $a,b\in {\mathbb {N}}$. It follows that

2.30 $$\begin{aligned} \sqrt{N}\beta _N u\big (\lceil Nt\rceil -\lceil Ns\rceil \big ) \le C \frac{1}{(t-s)^{1-\alpha '}}, \end{aligned}$$

for some $C \in (0,\infty )$, uniformly in $0 < s<t\le 1$ and $N\in {\mathbb {N}}$. Analogously, again by (2.10) and Potter bounds, if $0 \le s < t < \frac{r}{N}$ we have

2.31 $$\begin{aligned} \frac{u(\lceil N t\rceil -\lceil N s\rceil )}{u(r)} \le c^2 \frac{L(r+1)}{L(\lceil N t\rceil -\lceil N s\rceil + 1)} \frac{r^{1-\alpha }}{(N(t - s))^{1-\alpha }} \le C \frac{(r/N)^{1-\alpha '}}{(t-s)^{1-\alpha '}}.\nonumber \\ \end{aligned}$$

Plugging (2.30) and (2.31) into (2.28), and applying Lemma C.1, then gives

2.32 $$\begin{aligned} \Vert \widetilde{\Xi }_2^{(k)}\Vert _2^2&\le C^k \sum _{y=0}^{k-1} \mathop {\mathop {\idotsint }\limits _{0<t_1<\cdots <t_y<\frac{m}{N}}}\limits _ {\frac{m}{N}<t_{y+1}<\cdots <t_{k}<\frac{r}{N}}\frac{(r/N)^{2(1-\alpha ')}}{t_1^{2(1-\alpha ')} (t_2- t_1)^{2(1-\alpha ')}\cdots (r/N-t_k)^{2(1-\alpha ')}} \mathrm {d}t_1\cdots \mathrm {d}t_{k} \nonumber \\&\le C^k \sum _{y=0}^{k-1} C_1 e^{-C_2 k\log k} \Big (\frac{m}{N}\Big )^{(2\alpha '-1) y} \Big (\frac{r-m}{N}\Big )^{(2\alpha '-1)(k-y)} \nonumber \\&\le C_3 e^{-C_4k\log k} \Big (\frac{r-m}{N}\Big )^{2\alpha '-1}, \end{aligned}$$

where the last inequality follows by crude estimates (observe that $m/N \le 1$).

We can substitute the bound (2.32) into (2.27) to obtain

2.33 $$\begin{aligned} {\mathbb E} [|\widetilde{\Xi }_2|^{l}] \le \left( \sum _{k=1}^{r} (c_l)^k (C_3)^{\frac{1}{2}} e^{-\frac{C_4}{2}k\log k} \Big (\frac{r-m}{N}\Big )^{\alpha '-\frac{1}{2}} \right) ^l \le C \Big (\frac{r-m}{N}\Big )^{(\alpha '-\frac{1}{2}) l} \end{aligned}$$

for some $C$ depending only on $l$. We now choose $m$ as in (2.19), so that

$$\begin{aligned} \frac{r-m}{N}\le \frac{r-q + \sqrt{N(r-q)}}{N}\le 2\Big ( \frac{r-q}{N}\Big )^{\frac{1}{2}}, \end{aligned}$$

(if $m=0$ we first write $r=r-q+q$ and we use that in this case $q\le \sqrt{N(r-q)}$), hence

2.34 $$\begin{aligned} {\mathbb E} [|\widetilde{\Xi }_2|^l] \le C\, 2^{(\alpha '-\frac{1}{2}) l} \Big (\frac{r-q}{N}\Big )^{(\alpha '-\frac{1}{2}) \frac{l}{2}}. \end{aligned}$$

Since $\alpha '>\frac{1}{2}$ by our choice in (2.29), relation (2.25) is satisfied with $\eta = (\alpha '-\frac{1}{2}) \frac{l}{2}$ (and one has $\eta > 2 \frac{l}{l-1}$, as required, provided $l\in {\mathbb {N}}$ is chosen large enough).

Step 5: *bounding* ${\mathbb E} [|\widetilde{\Xi }_1|^{l}]$. Following the same steps as the bound for ${\mathbb E} [|\widetilde{\Xi }_2|^{l}]$, it suffices to establish an analogue of (2.32) for

2.35 $$\begin{aligned} \widetilde{\Xi }_1^{(k)} := \sum _{1\le n_1<\cdots < n_k\le m} \big (\psi _{N,r}(n_1,\ldots , n_k) -\psi _{N,q}(n_1,\ldots , n_k)\big ) \prod _{i=1}^k \widetilde{\zeta }_{N,n_i}, \end{aligned}$$

where we recall that $0\le m<q<r\le N$, because $m = m(q,r,N)$ is chosen as in (2.19). If $m=0$ then $\widetilde{\Xi }_1^{(k)} = 0$ and there is nothing to prove, hence we assume $m>0$ henceforth.

Since $(\widetilde{\zeta }_{N,i})_{i\in {\mathbb {N}}}$ are i.i.d. with ${\mathbb E} [\widetilde{\zeta }_{N,1}]=0$ and ${{\mathrm{\mathbb {V}ar}}}(\widetilde{\zeta }_{N,1})\le 1+C/\sqrt{N}\le 2$ for $N$ large,

2.36 $$\begin{aligned} \Vert \widetilde{\Xi }_1^{(k)}\Vert _2^2 = {\mathbb E} \big [\big (\widetilde{\Xi }_1^{(k)}\big )^2\big ] \le 2^k \sum _{1\le n_1<\cdots < n_k\le m} \big (\psi _{N,r}(n_1,\ldots , n_k)-\psi _{N,q} (n_1,\ldots , n_k)\big )^2 . \end{aligned}$$

Let $\varepsilon$ be as in condition (2.11). We first consider the case $r-q\ge \varepsilon ^2 r$, for which we bound

2.37 $$\begin{aligned} \Vert \widetilde{\Xi }_1^{(k)}\Vert _2^2&\le 2^{k+1} \sum _{1\le n_1<\cdots < n_k\le m} \big (\psi _{N,r}(n_1,\ldots , n_k)^2 + \psi _{N,q}(n_1,\ldots , n_k)^2\big ) \nonumber \\&\le 2^{k+1} \sum _{1\le n_1<\cdots < n_k\le r-1} \psi _{N,r}(n_1,\ldots , n_k)^2 \nonumber \\&+ 2^{k+1}\sum _{1\le n_1<\cdots < n_k\le q-1} \psi _{N,q}(n_1,\ldots , n_k)^2 . \end{aligned}$$

Applying the bound (2.32) with $m=0$, since $q < r$, we obtain

2.38 $$\begin{aligned} \Vert \widetilde{\Xi }_1^{(k)}\Vert _2^2 \le 2^{k+1} C_3e^{-C_4k\log k} \Big (\Big (\frac{r}{N}\Big )^{2\alpha '-1} + \Big (\frac{q}{N}\Big )^{2\alpha '-1}\Big ) \le C_5e^{-C_6 k\log k} \Big (\frac{r}{N}\Big )^{2\alpha '-1}, \end{aligned}$$

By the same calculation as in (2.33), we then have, using $r-q\ge \varepsilon ^2 r$,

$$\begin{aligned} {\mathbb E} [|\widetilde{\Xi }_1|^{l}] \le C \Big (\frac{r}{N}\Big )^{\left( \alpha '-\frac{1}{2}\right) l} \le \frac{C}{\varepsilon ^{(2\alpha ' - 1) l}} \Big (\frac{r-q}{N}\Big )^{\left( \alpha '-\frac{1}{2}\right) l}, \end{aligned}$$

which gives the desired bound (2.25).

Now we consider the case $r-q\le \varepsilon ^2 r$. Denote $I:=\{n_1, \ldots , n_k\}$. Recalling the definition of $\psi _{N,r}$ in (2.18), we have

2.39 $$\begin{aligned} \big (\psi _{N,r}(I)-\psi _{N,q}(I)\big )^2 = (\beta _N)^{2k} u(n_1)^2\cdots u(n_k-n_{k-1})^2 \bigg (\frac{u(r-n_k)}{u(r)} - \frac{u(q-n_k)}{u(q)}\bigg )^2.\nonumber \\ \end{aligned}$$

Since we assume $m > 0$, by (2.19) we have $m=q-\sqrt{N(r-q)}$ and $q, r\ge m+\sqrt{N}$. Recalling that $u(n)=\mathrm P(n\in \tau )$, we can bound the last factor in (2.39) as follows:

$$\begin{aligned}&\Big |\frac{\mathrm P(r-n_k\in \tau )}{\mathrm P(r\in \tau )} - \frac{\mathrm P(q-n_k\in \tau )}{\mathrm P(q\in \tau )}\Big |\\&\quad = \Big |\frac{\mathrm P(q\in \tau ) \mathrm P(r-n_k\in \tau ) - \mathrm P(r\in \tau )\mathrm P(q-n_k\in \tau )}{\mathrm P(q\in \tau )\mathrm P(r\in \tau )}\Big |\\&\quad = \bigg |\frac{\big [\mathrm P(q\!\in \!\tau )\!-\!\mathrm P(r\!\in \!\tau )\big ]\mathrm P(r\!-\!n_k\!\in \!\tau ) \!+\! \mathrm P(r\!\in \!\tau ) \big [\mathrm P(r\!-\!n_k\!\in \!\tau )\!-\!\mathrm P(q\!-\!n_k\!\in \!\tau )\big ]}{\mathrm P(q\!\in \!\tau )\mathrm P(r\!\in \!\tau )}\bigg | \\&\quad \le \frac{\big |\mathrm P(q\in \tau )-\mathrm P(r\in \tau )\big |\mathrm P(r-n_k\in \tau )}{\mathrm P(q\in \tau )\mathrm P(r\in \tau )}\\&\qquad \quad +\, \frac{\big |\mathrm P(q-n_k\in \tau )-\mathrm P(r-n_k\in \tau )\big |\mathrm P(q-n_k\in \tau )}{\mathrm P(q-n_k\in \tau )\mathrm P(q\in \tau )}. \end{aligned}$$

We now apply (2.11), using the assumption $r-q \le \varepsilon ^2 r<\varepsilon r$ and noting that

$$\begin{aligned} \frac{(r-n_k) - (q-n_k)}{r-n_k} \le \frac{r-q}{q-m} = \sqrt{\frac{r-q}{N}} \le \sqrt{\frac{r-q}{r}} \le \varepsilon , \end{aligned}$$

which yields

$$\begin{aligned} \bigg |\frac{u(r-n_k)}{u(r)} - \frac{u(q-n_k)}{u(q)}\bigg |&\le C \Big (\frac{r-q}{r}\Big )^\delta \, \frac{u(r-n_k)}{u(r)} + C \Big (\frac{r-q}{r-n_k}\Big )^\delta \, \frac{u(q-n_k)}{u(q)} \\&\le C \Big (\frac{r-q}{r}\Big )^\delta \frac{u(r-n_k)}{u(r)} + C \Big (\frac{r-q}{r}\Big )^{\delta /2} \frac{u(q-n_k)}{u(q)} . \end{aligned}$$

Plugging this into (2.39) and recalling (2.18) then gives

$$\begin{aligned} \big (\psi _{N,r}(I)-\psi _{N,q}(I)\big )^2&\le 2C^2 \Big (\frac{r-q}{r}\Big )^{2\delta } \psi _{N,r}(I)^2 + 2C^2 \Big (\frac{r-q}{r}\Big )^{\delta } \psi _{N,q}(I)^2 \\&\le 2C^2 \Big (\frac{r-q}{r}\Big )^{\delta } (\psi _{N,r}(I)^2+ \psi _{N,q}(I)^2). \end{aligned}$$

We can finally substitute this bound back into (2.36) and follow the same calculations as in (2.37)–(2.38), with an extra factor $\left( \frac{r-q}{r}\right) ^{\delta }$, to obtain

$$\begin{aligned} \Vert \widetilde{\Xi }_1^{(k)}\Vert _2^2 \le C_7 \, e^{-C_6 k\log k} \Big (\frac{r-q}{r}\Big )^{\delta } \Big (\frac{r}{N}\Big )^{2\alpha '-1} \le C_7 e^{-C_6 k\log k} \Big (\frac{r-q}{N}\Big )^{\delta \wedge (2\alpha '-1)}. \end{aligned}$$

By the same calculation as in (2.33), we then have

$$\begin{aligned} {\mathbb E} [|\widetilde{\Xi }_1|^{l}] \le C \Big (\frac{r-q}{N}\Big )^{\frac{\delta \wedge (2\alpha '-1)}{2} l}. \end{aligned}$$

Since $\delta >0$ and $\alpha '>1/2$, this gives the desired bound (2.25) for ${\mathbb E} [|\widetilde{\Xi }_1|^{l}]$, provided $l\in {\mathbb {N}}$ is chosen large enough. This completes the proof.$\square$

### Proof of Theorem 2.4

We fix $\alpha \in (1/2, 1)$ and $T \in (0,\infty )$. By Remark 1.2 and Lemma B.2 in the appendix, we can construct a renewal process $\tau$ satisfying (1.1), with $L(n)\rightarrow 1$ as $n\rightarrow \infty$, such that condition (2.11) is satisfied. By Theorem 2.1, for this particular renewal process, the discrete partition functions $\big (Z^{\omega ,\mathrm c}_{\beta _N,h_N}(sN, tN)\big )_{0\le s\le t\le T}$ converge in distribution as $N\rightarrow \infty$ to the continuum family $\big (\varvec{Z}^{\alpha ; W, \mathrm c}_{\hat{\beta }, \hat{h}}(s, t)\big )_{0\le s\le t\le T}$, viewed as random variables in $C([0,T]^2_\le , {\mathbb {R}})$. By Skorohod’s representation theorem [6, Thm. 6.7], we can couple $\big (Z^{\omega ,\mathrm c}_{\beta _N,h_N}\big )_{N\in {\mathbb {N}}}$ and $\varvec{Z}^{\alpha ; W, \mathrm c}_{\hat{\beta }, \hat{h}}$ so that, a.s., $Z^{\omega ,\mathrm c}_{\beta _N,h_N}(sN, tN)$ converges to $\varvec{Z}^{\alpha ; W, \mathrm c}_{\hat{\beta }, \hat{h}}(s, t)$ uniformly on $[0,T]^2_\le$. We assume such a coupling from now on.

Property (ii) is readily checked from the Wiener chaos representation (2.3). Alternatively, one can observe that similar properties hold for the disordered pinning partition functions $\big (Z^{\omega ,\mathrm c}_{\beta _N,h_N}(i, j)\big )_{0\le i\le j}$, which are preserved in the scaling limit.

We next prove (iv), where we may assume $0\le s<u<t\le T$. Let us fix a typical realization of $\big (Z^{\omega ,\mathrm c}_{\beta _N,h_N}\big )_{N\in {\mathbb {N}}}$ and $\varvec{Z}^{\alpha ; W, \mathrm c}_{\hat{\beta }, \hat{h}}$ under the above coupling. Let $a_N:= \lfloor sN\rfloor$, $b_N:=\lfloor uN\rfloor$ and $c_N:=\lfloor tN\rfloor$. Recalling the definition of $Z^{\omega , \mathrm c}_{\beta , h}$ in (1.21) and summing on the index $k\in {\mathbb {N}}$ for which $\tau _k < b_N \le \tau _{k+1}$ and on the values $i = \tau _k$, $j = \tau _{k+1}$, we obtain

2.40 $$\begin{aligned}&Z^{\omega , \mathrm c}_{\beta _N, h_N}(a_N,c_N) \mathrm P(c_N-a_N\in \tau ) \nonumber \\&\quad = \sum _{a_N\le i<b_N} \sum _{b_N\le j\le c_N} Z^{\omega , \mathrm c}_{\beta _N, h_N}(a_N,i) Z^{\omega , \mathrm c}_{\beta _N, h_N}(j,c_N) \ e^{(\beta _N \omega _i -\Lambda (\beta _N)+h_N)1\!\!1_{\{i>a_N\}}} \nonumber \\&\qquad \times \, \mathrm P(i-a_N\in \tau ) \mathrm P(\tau _1=j-i) \mathrm P(c_N-j\in \tau ) \ e^{(\beta _N \omega _j \!-\!\Lambda (\beta _N)\!+\!h_N)1\!\!1_{\{j<c_N\}}}.\nonumber \\ \end{aligned}$$

Multiply both sides of (2.40) by $N^{1-\alpha }$ and let $N\rightarrow \infty$. Since $\mathrm P(n\in \tau )\sim \frac{C_\alpha }{n^{1-\alpha }}$ by (2.10),

$$\begin{aligned} Z^{\omega , \mathrm c}_{\beta _N, h_N}(a_N,c_N) N^{1-\alpha } \mathrm P(c_N-a_N\in \tau ) \underset{{N}\rightarrow \infty }{\longrightarrow } \frac{C_\alpha \, \varvec{Z}^{\alpha ; W, \mathrm c}_{\hat{\beta }, \hat{h}}(s,t)}{(t-s)^{1-\alpha }} . \end{aligned}$$

For the RHS of (2.40), note that $\left( e^{(\beta _N \omega _i -\Lambda (\beta _N)+h_N)}\right) _{0\le i\le TN}$ converge uniformly to $1$ as $N\rightarrow \infty$ (because $\max \{ \omega _i: \, i \le TN\} = O(\log N)$ by Borel–Cantelli estimates, (1.3)). Moreover, for $i=\lfloor xN\rfloor$ and $j=\lfloor yN\rfloor$, with $s<x<u<y<t$,

$$\begin{aligned} Z^{\omega , \mathrm c}_{\beta _N, h_N}(a_N,\lfloor xN\rfloor ) Z^{\omega , \mathrm c}_{\beta _N, h_N}(\lfloor yN\rfloor ,c_N) \underset{{N}\rightarrow \infty }{\longrightarrow } \varvec{Z}^{\alpha ; W,\mathrm c}_{\hat{\beta }, \hat{h}}(s,x) \varvec{Z}^{\alpha ; W,\mathrm c}_{\hat{\beta }, \hat{h}}(y,t) \qquad \text{ uniformly }, \end{aligned}$$

while by $\mathrm P(\tau _1=n) = \frac{L(n)}{n^{1+\alpha }}$ and $\mathrm P(n\in \tau )\sim \frac{C_\alpha }{n^{1-\alpha }}$, (1.1) and (2.10), we get

2.41 $$\begin{aligned}&N^2 N^{1-\alpha } \mathrm P(\lfloor xN\rfloor -a_N\in \tau ) \mathrm P(\tau _1=\lfloor yN\rfloor -\lfloor xN\rfloor ) \mathrm P(c_N-\lfloor yN\rfloor \in \tau )\nonumber \\&\qquad \underset{{N}\rightarrow \infty }{\longrightarrow } \ \frac{C_\alpha ^2}{(x-s)^{1-\alpha } (y-x)^{1+\alpha } (t-y)^{1-\alpha }}, \end{aligned}$$

for all $s < x < u < y < t$ (the convergence is even uniform for $x-s, y-x, t-y\ge \varepsilon$, for any $\varepsilon >0$). Again by (1.1) and (2.10) with $L(n) \sim 1$, the LHS of (2.41) is uniformly bounded by a constant multiple of the RHS, which is integrable over $x\in (s,u)$ and $y\in (u,t)$. Therefore, by a Riemann sum approximation, the RHS of (2.40), multiplied by $N^{1-\alpha }$, converges to

$$\begin{aligned} \int _{x\in (s,u)} \int _{y\in (u,t)} \frac{C_\alpha \,\varvec{Z}^{\alpha ; W, c}_{\hat{\beta }, \hat{h}}(s,x)}{(x-s)^{1-\alpha }} \, \frac{1}{(y-x)^{1+\alpha }} \, \frac{C_\alpha \, \varvec{Z}^{\alpha ; W, c}_{\hat{\beta }, \hat{h}}(y,t)}{(t-y)^{1-\alpha }}\, \mathrm {d}x \, \mathrm {d}y, \end{aligned}$$

which establishes (2.8).

We then turn to (i), where we may restrict $s,t \in [0,T]$. The fact that $\varvec{Z}^{\alpha ; W, \mathrm c}_{\hat{\beta }, \hat{h}}(\cdot , \cdot )$ is a.s. continuous and non-negative follows readily from Theorem 2.1 [recall that $Z^{\omega ,\mathrm c}_{\beta _N,h_N}(i, j) \ge 0$, (1.21)]. For the a.s. strict positivity, we apply (2.8) with $u := (s+t)/2$: since $\varvec{Z}^{\alpha ; W, \mathrm c}_{\hat{\beta }, \hat{h}} \ge 0$, for any $\varepsilon > 0$ restricting the integrals to $x \le s+\varepsilon$ and $y \ge s-\varepsilon$ yields the lower bound

2.42 $$\begin{aligned} \varvec{Z}^{\alpha ; W, \mathrm c}_{\hat{\beta }, \hat{h}}(s,t) \ge (t-s)^{1-\alpha } \!\!\!\!\!\!\!\!\!\!\!\! \iint \limits _{x\in (s,(s+\varepsilon )\wedge t), \; y\in ((t-\varepsilon )\vee s,t)} \frac{C_\alpha \, \varvec{Z}^{\alpha ; W, \mathrm c}_{\hat{\beta }, \hat{h}}(s,x) \, \varvec{Z}^{\alpha ; W, \mathrm c}_{\hat{\beta }, \hat{h}}(y,t)}{(x\!-\!s)^{1-\alpha }(y-x)^{1+\alpha }(t-y)^{1-\alpha }}\, \mathrm {d}x \, \mathrm {d}y. \end{aligned}$$

Since $\varvec{Z}^{\alpha ; W, \mathrm c}_{\hat{\beta }, \hat{h}}(u,u) =1$ for all $u \ge 0$ (2.3), by continuity a.s. there is (a random) $\varepsilon >0$ such that $\varvec{Z}^{\alpha ; W, \mathrm c}_{\hat{\beta }, \hat{h}}(u,v)>0$ for all $u,v \in [0,T]$ with $0 \le v-u \le \varepsilon$. Observing that both $s-x \le \varepsilon$ and $y-t \le \varepsilon$ in (2.42) yields that a.s. $\varvec{Z}^{\alpha ; W, \mathrm c}_{\hat{\beta }, \hat{h}}(s,t) > 0$ for all $0 \le s \le t \le T$.

Lastly we prove (iii). For any $A>0$, recalling (2.3) and setting $\widetilde{W}_t := A^{-1/2}W_{At}$, the change of variables $t \mapsto u := t/A$ yields the equality in distribution (jointly in $s,t$)

$$\begin{aligned} \varvec{Z}^{\alpha ; W, \mathrm c}_{\hat{\beta }, \hat{h}}(As, At)&= 1 + \sum _{k=1}^\infty \ \ \idotsint \limits _{As<t_1<\cdots <t_k<At} \varvec{\psi }^{\alpha ; \mathrm c}_{As,At}(t_1,\ldots , t_k) \prod _{i=1}^k (\hat{\beta }\, \mathrm {d}W_{t_i} + \hat{h}\, \mathrm {d}t_i) \\&\qquad \overset{\mathrm {dist.}}{=} 1 + \sum _{k=1}^\infty \ \ \idotsint \limits _{s<u_1<\cdots < u_k<t} \varvec{\psi }^{\alpha ; \mathrm c}_{As,At}(A u_1,\ldots , A u_k) \\&\qquad \qquad \quad \times \, \prod _{i=1}^k (A^{1/2} \hat{\beta }\, \mathrm {d}\widetilde{W}_{u_i} + A\hat{h}\,\mathrm {d}u_i). \end{aligned}$$

Since $\varvec{\psi }^{\alpha ; \mathrm c}_{As,At}(A u_1,\ldots , A u_k) = A^{(\alpha -1) k} \varvec{\psi }^{\alpha ; \mathrm c}_{s,t}(u_1,\ldots , u_k)$, by (2.4), it follows that

$$\begin{aligned} \varvec{Z}^{\alpha ; W, \mathrm c}_{\hat{\beta }, \hat{h}}(As, At) \overset{\mathrm {dist.}}{=} \varvec{Z}^{\alpha ; \widetilde{W}, \mathrm c}_{A^{\alpha -1/2}\hat{\beta }, A^\alpha \hat{h}}(s, t). \end{aligned}$$

Since $\widetilde{W} = (\widetilde{W}_t)_{t\ge 0}$ is still a standard Brownian motion, the proof is completed. $\square$

## Characterization and universality of the CDPM

In this section we prove Theorems 1.3 and 1.6. We recall that ${\mathcal C}$ is the space of all closed subsets of ${\mathbb {R}}$, and refer to Appendix  for some key facts on ${\mathcal C}$-valued random variables (in particular for the notion of *restricted f.d.d.*, §). Let us summarize our setting:

- we have two independent sources of randomness: a renewal process $\tau = (\tau _n)_{n\ge 0}$ satisfying (1.1) and (1.7), and an i.i.d. sequence $\omega = (\omega _n)_{n\ge 1}$ satisfying (1.3);
- we fix $T > 0$ and consider the *conditioned pinning model* $\mathrm P^{\omega , \mathrm c}_{NT,\beta ,h}$, defined in (1.9) and (1.4), with the parameters $\beta = \beta _N$ and $h = h_N$ chosen as in (1.11).

Let us denote by $X_N$ the rescaled set $\tau /N \cap [0,T]$ (1.2), under the law $\mathrm P^{\omega , \mathrm c}_{NT, \beta _N ,h_N}$. If we *fix* a realization of $\omega$, then $X_N$ is a ${\mathcal C}$-valued random variable (with respect to $\tau$).

Our strategy to prove Theorems 1.3 and 1.6 is based on two main steps:

1. first we define a suitable coupling of $\omega$ with a standard Brownian motion $W$;
2. then we show that, for ${\mathbb P}$-a.e. *fixed* realization of $(\omega , W)$, the restricted f.d.d. of $X_N$ converge weakly as $N\rightarrow \infty$ to those given in the right hand side of (1.22).

We can then apply Proposition A.6 (iii), which guarantees that the densities in (1.22) are the restricted f.d.d. of a ${\mathcal C}$-valued random variable $X_\infty$, whose law on ${\mathcal C}$ we denote by $\varvec{\mathrm {P}}^{\alpha ; W, \mathrm c}_{T, \hat{\beta }, \hat{h}}$; furthermore, $X_N$ converges in distribution on ${\mathcal C}$ toward $X_\infty$ as $N\rightarrow \infty$, for ${\mathbb P}$-a.e. *fixed* realization of $(\omega , W)$. This is nothing but Theorem 1.3 in a strengthened form, with a.s. convergence instead of convergence in distribution (thanks to the coupling). Theorem 1.6 is also proved, once we note that $\varvec{\mathrm {P}}^{\alpha ; W, \mathrm c}_{T, \hat{\beta }, \hat{h}}$ is the *unique* probability law on ${\mathcal C}$ satisfying conditions (i) and (ii) therein, because restricted f.d.d. characterize laws on ${\mathcal C}$, Proposition A.6 (i). It only remains to prove points (1) and (2).

By Theorem 2.1, the family $Z_N := \big (Z^{\omega ,\mathrm c}_{\beta _N,h_N}(sN, tN)\big )_{0\le s\le t\le T}$ of discrete partition functions defined in (1.21), viewed as a $C([0,T]^2_\le , {\mathbb {R}})$-valued random variable, converges in distribution to the continuum family $\varvec{Z}:= \big (\varvec{Z}^{\alpha ; W, \mathrm c}_{\hat{\beta }, \hat{h}}(s, t)\big )_{0\le s\le t\le T}$ as $N\rightarrow \infty$. Note that $Z_N$ is a function of $\omega _{(0,N)} := (\omega _1, \ldots , \omega _{N-1})$, while $\varvec{Z}$ is a function of a standard Brownian motion $W = (W_t)_{t\ge 0}$. By an extension of Skorohod’s representation theorem [23, Cor. 5.12], we can couple the discrete environments $(\omega _{(0,N)})_{N\in {\mathbb {N}}}$ and $W$ on the same probability space, so that $Z_N \rightarrow \varvec{Z}$ a.s.. This completes point (1).

Coming to point (2), we prove the convergence of the restricted f.d.d. of $X_N$ , i.e. the laws of the vectors $(\mathtt g_{t_1}(X_N), \ldots , \mathtt g_{t_k}(X_N))$ restricted on the event $A_{t_1,\ldots , t_k}^{X_N}$ defined in (4.16). Since $X_N = \tau /N \cap [0,T]$ under the pinning law $\mathrm P_{TN,\beta _N,h_N}^{\omega , \mathrm c}$, we fix $k \in {\mathbb {N}}$ and $0 < t_1 < \ldots < t_k < T$, as well as a continuous and bounded function $F: {\mathbb {R}}^{2k} \rightarrow {\mathbb {R}}$, and we have to show that

3.1 $$\begin{aligned} I_N := \mathrm E_{TN,\beta _N,h_N}^{\omega , \mathrm c} \Big [ F\big (\mathtt g_{t_1}(\tau /N), \, \mathtt d_{t_1}(\tau /N), \ldots , \mathtt g_{t_k}(\tau /N), \mathtt d_{t_k}(\tau /N)\big ) 1\!\!1_{A_{t_1,\ldots , t_k}^{\tau /N}} \Big ]\nonumber \\ \end{aligned}$$

converges as $N\rightarrow \infty$ to the integral of $F$ with respect to the density in (1.22), i.e.

3.2 $$\begin{aligned} I&:= \mathop {\mathop {\idotsint }\limits _{0<x_1<t_1<y_1<x_2<t_2}}\limits _{<\cdots <x_k<t_k<y_k<T} F(x_1,y_1,\ldots , x_k, y_k) \times \Bigg \{\, \frac{\prod _{i=0}^k \varvec{Z}^{\alpha ; W, \mathrm c}_{\hat{\beta }, \hat{h}}(y_{i},x_{i+1})}{\varvec{Z}^{\alpha ; W, \mathrm c}_{\hat{\beta }, \hat{h}}(0,T)} \Bigg \}\,\nonumber \\&\times \, \Bigg [ \prod _{i=1}^{k} \frac{C_\alpha }{(x_{i} - y_{i-1})^{1-\alpha } (y_i-x_i)^{1+\alpha }} \Bigg ] \frac{T^{1-\alpha }}{(T-y_k)^{1-\alpha }} \, \mathrm {d}x \, \mathrm {d}y, \end{aligned}$$

where we set $y_0 := 0$, $x_{k+1} := T$ and $\mathrm {d}x \, \mathrm {d}y$ is a shorthand for $\mathrm {d}x_1 \mathrm {d}y_1 \cdots \mathrm {d}x_k \mathrm {d}y_k$.

Denoting by $\mathrm P_{N}^\mathrm{c}$ the law of the renewal process $\tau \cap [0,N]$ conditioned to visit $N$,

3.3 $$\begin{aligned} \mathrm P_N^\mathrm{c}(\,\cdot \,) := \mathrm P( \tau \cap [0,N] \in \,\cdot \,|\, N \in \tau ), \end{aligned}$$

the pinning law $\mathrm P^{\omega , \mathrm c}_{N,\beta ,h}$ can be written as follows (1.4), (1.9) and (1.21):

3.4 $$\begin{aligned} \frac{\mathrm P^{\omega , \mathrm c}_{N,\beta ,h}(\tau )}{\mathrm P_N^\mathrm{c}(\tau )} := \frac{1}{Z^{\omega , \mathrm c}_{\beta ,h}(0,N)} e^{\sum _{n=1}^{N-1} (\beta \omega _n -\Lambda (\beta ) + h) 1\!\!1_{\{n\in \tau \}}}. \end{aligned}$$

In particular, the law $\mathrm P^{\omega , \mathrm c}_{TN,\beta ,h}$ reduces to $\mathrm P_{TN}^\mathrm{c}$ for $\beta = h = 0$. In this special case, the convergence $I_N \rightarrow I$ is shown in the proof of Proposition A.8, (4.23) and the following lines, exploiting the renewal decomposition (4.25) for $I_N$. In the general case, with $\mathrm P^{\omega , \mathrm c}_{TN,\beta ,h}$ instead $\mathrm P_{TN}^\mathrm{c}$, we have a completely analogous decomposition, thanks to (3.4):

$$\begin{aligned} I_N&= \frac{1}{N^{2k}} \sum _{0 \le a_1 \le Nt_1} \, \sum _{N t_1 < b_1 \le a_2 \le Nt_2} \cdots \sum _{N t_{k-1} < b_{k-1} \le a_k \le Nt_k}\sum _{N t_k < b_k \le NT} \\&\times F\Big (\frac{a_1}{N}, \frac{b_1}{N}, \ldots , \frac{a_k}{N}, \frac{b_k}{N}\Big ) \\&\times \, \left\{ \prod _{i=1}^k e^{\beta _N (\omega _{a_i} + \omega _{b_i}) - 2 \Lambda (\beta _N) + 2 h_N}\right\} \left\{ \, \frac{\prod _{i=0}^k Z^{\omega , \mathrm c}_{\beta _N, h_N}(b_{i},a_{i+1})}{Z^{\omega , \mathrm c}_{\beta _N, h_N}(0,NT)} \right\} \, \\&\times \, \left[ \prod _{i=1}^{k} N^2 \, u(a_i - b_{i-1}) K(b_i - a_i) \right] \frac{u(\lfloor NT\rfloor -b_k)}{u(\lfloor NT\rfloor )}. \end{aligned}$$

We stress that the difference with respect to (4.25) is only given by the two terms in brackets appearing in the middle line. The first term in brackets converges to $1$ as $N\rightarrow \infty$, because $\max _{0 \le n \le NT} |\omega _n| = O(\log N)$ (as we already remarked in §2.3). When we set $a_i = N x_i$ and $b_i = N y_i$, the second term in brackets converges to its analogue in (3.2) involving the continuum partition functions, for ${\mathbb P}$-a.e. fixed realization of $(\omega ,W)$ (thanks to our coupling), and is uniformly bounded by some (random) constant, because the continuum partition functions are a.s. continuous and strictly positive Theorem 2.4 (i). Since the convergence of (4.23)–(4.24) is shown by a Riemann sum approximation, the convergence of (3.1)– (3.2) follows immediately, completing the proof of point (2). $\square$

## Key properties of the CDPM

In this section, we prove Theorems 1.4 and 1.5. The parameters $\alpha \in \left( \frac{1}{2}, 1\right)$, $T > 0$, ${\hat{\beta }}> 0$ and ${\hat{h}}\in {\mathbb {R}}$ are fixed throughout the section. We use in an essential way the continuum partition functions $\big (\varvec{Z}^{\alpha ; W, \mathrm c}_{\hat{\beta }, \hat{h}}(s, t)\big )_{0\le s\le t\le T}$ Theorems 2.1 and 2.4, and the characterization of the CDPM quenched law $\varvec{\mathrm {P}}^{\alpha ; W, \mathrm c}_{T, \hat{\beta }, {\hat{h}}}$ in terms of restricted f.d.d., given in Theorem 1.6.

### *Proof of Theorem 1.4*

Assume that ${\hat{h}}= 0$, and recall definition (1.13) of the “reference law” $\varvec{\mathrm {P}}^{\alpha ; \mathrm c}_T$. We will show at the end of the proof the following equality of two probability measures on ${\mathcal C}$:

4.1 $$\begin{aligned} {\mathbb E} \big [ \varvec{Z}^{\alpha ; W, \mathrm c}_{\hat{\beta },0}(0,T) \, \varvec{\mathrm {P}}^{\alpha ; W, \mathrm c}_{T, \hat{\beta }, 0} (\cdot ) \big ] = \varvec{\mathrm {P}}^{\alpha ; \mathrm c}_T(\cdot ). \end{aligned}$$

Let us assume this for the moment.

By (4.1), if $\varvec{\mathrm {P}}^{\alpha ; \mathrm c}_T(A) = 0$ for some $A \subseteq {\mathcal C}$, then $\varvec{\mathrm {P}}^{\alpha ; W, \mathrm c}_{T, \hat{\beta }, 0} (A) = 0$ for ${\mathbb P}$-a.e. $W$ (because $\varvec{Z}^{\alpha ; W, \mathrm c}_{\hat{\beta },0}(0,T)>0$ a.s., by Theorem 2.4 (i)), hence ${\mathbb E} [\varvec{\mathrm {P}}^{\alpha ; W, \mathrm c}_{T, \hat{\beta }, 0} (A)] = 0$. This shows that the law ${\mathbb E} [\varvec{\mathrm {P}}^{\alpha ; W, \mathrm c}_{T, \hat{\beta }, 0}(\cdot )]$ is absolutely continuous with respect to $\varvec{\mathrm {P}}^{\alpha ; \mathrm c}_T(\cdot )$, proving Theorem 1.4 for ${\hat{h}}= 0$.

We now turn to the case ${\hat{h}}\ne 0$. By Remark 2.2, the continuum partition functions $\big (\varvec{Z}^{\alpha ; W, \mathrm c}_{\hat{\beta }, \hat{h}}(s, t)\big )_{0\le s\le t\le T}$ have a law that, for ${\hat{h}}\ne 0$, is absolutely continuous with respect to case ${\hat{h}}= 0$, with Radon-Nikodym density $\mathfrak {f}_{T, {\hat{\beta }}, {\hat{h}}}(W)$ given in (2.5). Since the restricted f.d.d. of $\varvec{\mathrm {P}}^{\alpha ; W, \mathrm c}_{T, \hat{\beta }, \hat{h}}$ are expressed in terms of continuum partition functions (1.22), the two probability measures ${\mathbb E} [\varvec{\mathrm {P}}^{\alpha ; W, \mathrm c}_{T, \hat{\beta }, {\hat{h}}}(\cdot )]$ and ${\mathbb E} [ \mathfrak {f}_{T, {\hat{\beta }}, {\hat{h}}}(W) \, \varvec{\mathrm {P}}^{\alpha ; W, \mathrm c}_{T, \hat{\beta }, 0}(\cdot )]$ on ${\mathcal C}$ have the same restricted f.d.d. and hence are identical, by Proposition A.6 (i). As a consequence, if ${\mathbb E} [\varvec{\mathrm {P}}^{\alpha ; W, \mathrm c}_{T, \hat{\beta }, {\hat{h}}} (A)] = 0$ for ${\hat{h}}= 0$, the same is true also for ${\hat{h}}\ne 0$, completing the proof of Theorem 1.4.

It remains to establish (4.1). Note that its LHS is indeed a probability law on ${\mathcal C}$, since ${\mathbb E} [\varvec{Z}^{\alpha ; W, \mathrm c}_{\hat{\beta },0}(0,T)]=1$ by the Wiener-chaos expansion in (2.3) with $\hat{h}=0$. It suffices to show that the LHS and RHS in (4.1) have the same restricted f.d.d., by Proposition A.6 (i), and this follows immediately from relations (1.19) and (1.22), because ${\mathbb E} [\varvec{Z}^{\alpha ; W, \mathrm c}_{\hat{\beta }, 0}(y_{i},x_{i+1})] = 1$.

Lastly, we note that the $\alpha$-stable regenerative set $\varvec{\tau }$ a.s. has Hausdorff dimension $\alpha$ (see e.g. [5, Thm. III.15]), and the same holds a.s. under the conditioned measure $\varvec{\mathrm {P}}^{\alpha ; \mathrm c}_T$, which then carries over to the quenched law $\varvec{\mathrm {P}}^{\alpha ; W, \mathrm c}_{T, {\hat{\beta }}, {\hat{h}}}$ of the CDPM, for a.e. realization of $W$. $\square$

### *Proof of Theorem 1.5*

Let us set

$$\begin{aligned} {\mathcal C} _{0,T} := \{K\in {\mathcal C} : 0,T \in K \subset [0,T]\}. \end{aligned}$$

By construction, $\varvec{\mathrm {P}}^{\alpha ; W, \mathrm c}_{T, {\hat{\beta }}, 0}$ and $\varvec{\mathrm {P}}^{\alpha ; \mathrm c}_T$ are probability measures on ${\mathcal C} _{0,T}$, equipped with the Borel $\sigma$-algebra ${\mathcal F}$. Recalling the definition (1.16) of the maps $\mathtt g_t, \mathtt d_t$, for $n\in {\mathbb {N}}$ let ${\mathcal F} _n$ be the $\sigma$-algebra on ${\mathcal C} _{0,T}$ generated by $\mathtt g_{\frac{i}{2^n}T}$ and $\mathtt d_{\frac{i}{2^n}T}$ for $1\le i\le 2^n-1$. Then $({\mathcal F} _n)_{n\in {\mathbb {N}}}$ is a filtration on ${\mathcal C} _{0,T}$ that generates the Borel $\sigma$-algebra ${\mathcal F}$ on ${\mathcal C} _{0,T}$, by Lemma A.2.

Let $f^W_n: {\mathcal C} _{0,T} \rightarrow [0,\infty ]$ be the Radon-Nikodym derivative of $\varvec{\mathrm {P}}^{\alpha ; W, \mathrm c}_{T, {\hat{\beta }}, {\hat{h}}}$ with respect to $\varvec{\mathrm {P}}^{\alpha ; \mathrm c}_T$ on $({\mathcal C} _{0,T}, {\mathcal F} _n)$. If $\varvec{\tau }$ is a ${\mathcal C} _{0,T}$-valued random variable with law $\varvec{\mathrm {P}}^{\alpha ; \mathrm c}_T$, then $(f^W_n(\varvec{\tau }))_{n\in {\mathbb {N}}}$ is a non-negative martingale adapted to the filtration $({\mathcal F} _n)_{n\in {\mathbb {N}}}$, and $\varvec{\mathrm {P}}^{\alpha ; W, \mathrm c}_{T, {\hat{\beta }}, {\hat{h}}}$ is singular w.r.t. $\varvec{\mathrm {P}}^{\alpha ; \mathrm c}_T$ if and only if $f^W_n(\varvec{\tau }) \rightarrow 0$ a.s.. To prove Theorem 1.5, it suffices to show that, under the joint law of $\varvec{\tau }$ and $W$,

4.2 $$\begin{aligned} f^W_n(\varvec{\tau }) \xrightarrow [n\rightarrow \infty ]{} 0 \quad \ \ \text {in probability} \,, \end{aligned}$$

because we already know that the martingale limit $\lim _{n\rightarrow \infty } f^W_n(\varvec{\tau })$ exists a.s..

We next identify $f^W_n(\varvec{\tau })$. Without loss of generality, assume $T=1$. To remove duplicates among the random variables $\mathtt g_{\frac{i}{2^{n}}}$, $\mathtt d_{\frac{i}{2^{n}}}$, for $1\le i\le 2^n-1$, let us set

$$\begin{aligned}&I_n(\varvec{\tau }) := \left\{ 2\le i\le 2^n - 1: \ \varvec{\tau }\cap \Big (\tfrac{i-1}{2^{n}}, \tfrac{i}{2^{n}}\Big ] \ne \emptyset \right\} ,\\&a_{n,j}(\varvec{\tau }) := \min \left\{ \varvec{\tau }\cap \left[ \tfrac{j-1}{2^{n}}, \tfrac{j}{2^{n}}\right] \right\} , \quad \ b_{n,j}(\varvec{\tau }) := \max \left\{ \varvec{\tau }\cap \Big [\tfrac{j-1}{2^{n}}, \tfrac{j}{2^{n}}\Big ] \right\} . \end{aligned}$$

Then we claim that the following explicit expression for $f_n^W(\varvec{\tau })$ holds:

4.3 $$\begin{aligned} f_n^W(\varvec{\tau }) = \frac{\prod _{j \in \{1\} \cup I_n(\varvec{\tau }) \cup \{2^n\}} \varvec{Z}^{\alpha ; W, \mathrm c}_{{\hat{\beta }},{\hat{h}}}(a_{n,j}(\varvec{\tau }), b_{n,j}(\varvec{\tau }))}{\varvec{Z}^{\alpha ; W, \mathrm c}_{{\hat{\beta }},{\hat{h}}}(0,1)} \,. \end{aligned}$$

In order to prove it, first note that $f_n^W(\varvec{\tau })$ must necessarily be a function of the vector

4.4 $$\begin{aligned} V_n(\varvec{\tau }) := \Big ( \mathtt g_{\frac{1}{2^n}}(\varvec{\tau }), \, \big (\mathtt d_{\frac{j-1}{2^n}}(\varvec{\tau }) , \mathtt g_{\frac{j}{2^n}}(\varvec{\tau })\big )_{j \in I_n(\varvec{\tau })}, \, \mathtt d_{1-\frac{1}{2^n}}(\varvec{\tau }) \Big ) \,, \end{aligned}$$

because the variables $\mathtt g_{\frac{i}{2^{n}}}(\varvec{\tau })$, $\mathtt d_{\frac{i}{2^{n}}}(\varvec{\tau })$ for $i\not \in I_n(\varvec{\tau })$ are just repetitions of those in $V_n(\varvec{\tau })$.^3^ Then observe that $V_n(\varvec{\tau })$ can be rewritten equivalently as

$$\begin{aligned} V_n(\varvec{\tau }) = \Big ( \mathtt g_{\frac{1}{2^n}}(\varvec{\tau }), \, \mathtt d_{\frac{1}{2^n}}(\varvec{\tau }), \, \big (\mathtt g_{\frac{j}{2^n}}(\varvec{\tau }), \mathtt d_{\frac{j}{2^n}}(\varvec{\tau })\big )_{j \in I_n(\varvec{\tau })} \Big ) \,, \end{aligned}$$

because if $j < j'$ are consecutive points in $I_n(\varvec{\tau })$ then $\mathtt d_{\frac{j}{2^n}}(\varvec{\tau }) = \mathtt d_{\frac{j'-1}{2^n}}(\varvec{\tau })$. Finally, note that on the event $I_n(\varvec{\tau }) = J$, defining $t_1 := \frac{1}{2^n}$ and $\{t_2, \ldots , t_k\} := \{\frac{j}{2^n}\}_{j\in J}$, the density of the random vector $V_n(\varvec{\tau })$ (w.r.t. Lebesgue measure on ${\mathbb {R}}^{2k}$) under $\varvec{\mathrm {P}}^{\alpha ; \mathrm c}_1$ is given by (1.19), while under $\varvec{\mathrm {P}}^{\alpha ; W, \mathrm c}_{1, {\hat{\beta }}, {\hat{h}}}$ is given by (1.22); then (4.3) follows comparing (1.19) and (1.22).

Since $\varvec{Z}^{\alpha ; W, \mathrm c}_{{\hat{\beta }},0}(0,1)>0$ a.s. by Theorem 2.4, relation (4.2) will follow by showing that

4.5 $$\begin{aligned} \varvec{\mathrm {P}}^{\alpha ; \mathrm c}_1-\hbox {a.s.}, \qquad {\mathbb E} \big [\big (f^W_n(\varvec{\tau }) \varvec{Z}^{\alpha ; W, \mathrm c}_{{\hat{\beta }}, {\hat{h}}}(0,1)\big )^\gamma \big ] \underset{{n}\rightarrow \infty }{\longrightarrow } 0 \qquad \hbox {for some } \gamma \in (0,1). \end{aligned}$$

We can reduce to the case ${\hat{h}}= 0$ using Remark 2.2, in particular (2.5), writing

$$\begin{aligned} {\mathbb E} \big [\big (f^W_n(\varvec{\tau }) \varvec{Z}^{\alpha ; W, \mathrm c}_{{\hat{\beta }}, {\hat{h}}}(0,1)\big )^\gamma \big ]&= {\mathbb E} \big [ \mathfrak {f}_{1, {\hat{\beta }}, {\hat{h}}}(W) \big (f^W_n(\varvec{\tau }) \varvec{Z}^{\alpha ; W, \mathrm c}_{{\hat{\beta }}, 0}(0,1)\big )^\gamma \big ] \\&\le {\mathbb E} \big [ \mathfrak {f}_{1, {\hat{\beta }}, {\hat{h}}}(W)^{\frac{p}{p-1}}\big ]^{\frac{p-1}{p}} \, {\mathbb E} \big [ \big (f^W_n(\varvec{\tau }) \varvec{Z}^{\alpha ; W, \mathrm c}_{{\hat{\beta }}, 0}(0,1) \big )^{\gamma p} \big ]^{1/p} \,. \end{aligned}$$

Choosing $p \in (1,\infty )$ close to $1$, it suffices to prove (4.5) in the special case ${\hat{h}}= 0$.

Henceforth we fix ${\hat{h}}= 0$. For a given realization of $\varvec{\tau }$, we note that the factors in the numerator of (4.3) are independent, by Theorem 2.4 (i). Therefore

4.6 $$\begin{aligned} {\mathbb E} \big [\big (f^W_n(\varvec{\tau }) \varvec{Z}^{\alpha ; W, \mathrm c}_{{\hat{\beta }},0}(0,1)\big )^\gamma \big ]&= \prod _{j\in \{1\} \cup I_n(\varvec{\tau }) \cup \{2^n\}} {\mathbb E} \big [\big ( \varvec{Z}^{\alpha ; W, \mathrm c}_{{\hat{\beta }},0} (a_{n,j}(\varvec{\tau }), b_{n,j}(\varvec{\tau })) \big )^\gamma \big ] \nonumber \\&= \prod _{j\in \{1\} \cup I_n(\varvec{\tau }) \cup \{2^n\}} {\mathbb E} \big [\big ( \varvec{Z}^{\alpha ; W, \mathrm c}_{{\hat{\beta }}_{n,j}(\varvec{\tau }),0}(0,1) \big )^\gamma \big ], \end{aligned}$$

where we set ${\hat{\beta }}_{n,j}(\varvec{\tau }) := {\hat{\beta }}(b_{n,j}(\varvec{\tau })-a_{n,j}(\varvec{\tau }))^{\alpha -1/2}$ and we used the translation invariance and scaling property of $\varvec{Z}^{\alpha ; W, \mathrm c}_{{\hat{\beta }},0}(\cdot , \cdot )$ established in Theorem 2.4 (ii)–(iii).

We claim that for any $\gamma \in \left( 0,\frac{1}{2}\right)$ there exists $c=c(\gamma )>0$ such that for ${\hat{\beta }}>0$ sufficiently small,

4.7 $$\begin{aligned} {\mathbb E} \big [\big ( \varvec{Z}^{\alpha ; W, \mathrm c}_{{\hat{\beta }},0}(0,1) \big )^\gamma \big ] \le 1-c {\hat{\beta }}^2 \le e^{-c{\hat{\beta }}^2}. \end{aligned}$$

Substituting this bound into (4.6) then gives

$$\begin{aligned} \log {\mathbb E} \big [\big (f^W_n(\varvec{\tau }) \varvec{Z}^{\alpha ; W, \mathrm c}_{{\hat{\beta }},0}(0,1)\big )^\gamma \big ] \le - c{\hat{\beta }}^2 \!\!\!\!\sum _{j\in \{1\} \cup I_n(\varvec{\tau }) \cup \{2^n\}}\!\!\!\! (b_{n,j}(\varvec{\tau })-a_{n,j}(\varvec{\tau }))^{2\alpha -1}. \end{aligned}$$

The RHS diverges $\varvec{\mathrm {P}}^{\alpha ; \mathrm c}_1(\mathrm {d}\varvec{\tau })$-a.s. as $n\rightarrow \infty$, because $\{[a_{n,j}(\varvec{\tau }), b_{n,j}(\varvec{\tau })]\}_{j\in \{1\} \cup I_n\cup \{2^{n}\}}$ is a covering of $\varvec{\tau }$ with balls of diameter at most $2^{-n}$, and $\varvec{\tau }$ a.s. has Hausdorff dimension $\alpha$, which is strictly larger than $2\alpha -1$ for $\alpha \in (\frac{1}{2},1)$. The divergence follows from the definition of the Hausdorff dimension (see e.g. [5, Section III.5].

Lastly we prove (4.7). By (2.3), we have the representation

$$\begin{aligned} \varvec{Z}^{\alpha ; W, \mathrm c}_{{\hat{\beta }}, 0}(0,1) = 1 + \sum _{k=1}^\infty {\hat{\beta }}^k Y_k \,, \end{aligned}$$

where $Y_k$ is a random variable in the $k$-th order Wiener chaos expansion, and we recall that the series converges in $L^2$ for all ${\hat{\beta }}>0$. By Taylor expansion, there exist $\varepsilon , C>0$ such that

$$\begin{aligned} (1+x)^\gamma \le 1+ \gamma x - Cx^2 \qquad \text{ for } \text{ all } |x|\le \varepsilon . \end{aligned}$$

For later convenience, let us define

$$\begin{aligned} {\mathcal S} _{\hat{\beta }}:= \sum _{k=1}^\infty {\hat{\beta }}^k Y_k \,, \qquad {\mathcal T} _{\hat{\beta }}:= \sum _{k=2}^\infty {\hat{\beta }}^k Y_k \,. \end{aligned}$$

We then obtain

4.8 $$\begin{aligned}&{\mathbb E} \big [\big ( \varvec{Z}^{\alpha ; W, \mathrm c}_{{\hat{\beta }},0} (0,1) \big )^\gamma \big ] = {\mathbb E} \big [(1+{\mathcal S} _{\hat{\beta }})^\gamma 1\!\!1_{\{|{\mathcal S} _{\hat{\beta }}|\le \varepsilon \}}\big ] + {\mathbb E} \big [(1+{\mathcal S} _{\hat{\beta }})^\gamma 1\!\!1_{\{|{\mathcal S} _{\hat{\beta }}|> \varepsilon \}}\big ] \nonumber \\&\quad \le 1+ \gamma {\mathbb E} [{\mathcal S} _{\hat{\beta }}1\!\!1_{\{|{\mathcal S} _{\hat{\beta }}|\le \varepsilon \}}] - C{\mathbb E} [{\mathcal S} _{\hat{\beta }}^21\!\!1_{\{|{\mathcal S} _{\hat{\beta }}|\le \varepsilon \}}] + {\mathbb E} [1+{\mathcal S} _{\hat{\beta }}]^\gamma {\mathbb P} (|{\mathcal S} _{\hat{\beta }}|\ge \varepsilon )^{1-\gamma } \nonumber \\&\quad = 1 - C {\mathbb E} [{\mathcal S} _{\hat{\beta }}^2] + \big \{\!- \gamma {\mathbb E} [{\mathcal S} _{\hat{\beta }}1\!\!1_{\{|{\mathcal S} _{\hat{\beta }}| > \varepsilon \}}] + C{\mathbb E} [{\mathcal S} _{\hat{\beta }}^21\!\!1_{\{|{\mathcal S} _{\hat{\beta }}| > \varepsilon \}}] + {\mathbb P} (|{\mathcal S} _{\hat{\beta }}|\ge \varepsilon )^{1-\gamma } \big \},\nonumber \\ \end{aligned}$$

having used the fact that ${\mathbb E} [{\mathcal S} _{\hat{\beta }}] = 0$ in the last line. Observe that

$$\begin{aligned} {\mathbb E} [{\mathcal S} _{\hat{\beta }}^2] = \sum _{k=1}^\infty {\hat{\beta }}^{2k} \mathrm E[Y_k^2] = \mathrm E[Y_1^2] {\hat{\beta }}^2 + O({\hat{\beta }}^4) \qquad \text {as } {\hat{\beta }}\downarrow 0 \,, \end{aligned}$$

hence the first two terms in (4.8) give the correct asymptotic behavior (4.7). It remains to show that the three terms in brackets are $o({\hat{\beta }}^2)$. Note that

$$\begin{aligned} {\mathbb E} [{\mathcal T} _{\hat{\beta }}^2] = \sum _{k=2}^\infty {\hat{\beta }}^{2k} \mathrm E[Y_k^2] = O({\hat{\beta }}^4) \,, \end{aligned}$$

and moreover ${\mathbb E} [Y_1^4] \le (const.) {\mathbb E} [Y_1^2]^2 < \infty$, by the hyper-contractivity of Wiener chaos expansions (see e.g. [22, Thm. 3.50]). Writing ${\mathcal S} _{\hat{\beta }}= {\hat{\beta }}Y_1 + {\mathcal T} _{\hat{\beta }}$, we obtain

$$\begin{aligned} {\mathbb P} (|{\mathcal S} _{\hat{\beta }}| \!\ge \! \varepsilon ) \le {\mathbb P} (|{\hat{\beta }}Y_1| \ge \tfrac{1}{2}\varepsilon ) + {\mathbb P} (|{\mathcal T} _{\hat{\beta }}| \ge \tfrac{1}{2} \varepsilon ) \le \big (\tfrac{2}{\varepsilon }\big )^4 \mathrm E[Y_1^4] {\hat{\beta }}^4 + \big (\tfrac{2}{\varepsilon }\big )^2 {\mathbb E} [{\mathcal T} _{\hat{\beta }}^2] \!= \!O({\hat{\beta }}^4). \end{aligned}$$

Since ${\mathbb E} [{\mathcal S} _{\hat{\beta }}^2] = O({\hat{\beta }}^2)$, we can also write

$$\begin{aligned} \big | {\mathbb E} [{\mathcal S} _{\hat{\beta }}1\!\!1_{\{|{\mathcal S} _{\hat{\beta }}| > \varepsilon \}}] \big | \le {\mathbb E} [{\mathcal S} _{\hat{\beta }}^2]^{1/2} \, {\mathbb P} (|{\mathcal S} _{\hat{\beta }}| > \varepsilon )^{1/2} = O({\hat{\beta }}^3), \end{aligned}$$

and analogously

$$\begin{aligned} {\mathbb E} [{\mathcal S} _{\hat{\beta }}^21\!\!1_{\{|{\mathcal S} _{\hat{\beta }}| > \varepsilon \}}]&\le 2 {\mathbb E} [({\hat{\beta }}Y_1)^21\!\!1_{\{|{\mathcal S} _{\hat{\beta }}| > \varepsilon \}}] + 2 {\mathbb E} [{\mathcal T} _{\hat{\beta }}^21\!\!1_{\{|{\mathcal S} _{\hat{\beta }}| > \varepsilon \}}] \\&\le 2 {\hat{\beta }}^2 {\mathbb E} [Y_1^4]^{1/2} \, {\mathbb P} (|{\mathcal S} _{\hat{\beta }}| > \varepsilon )^{1/2} + 2 {\mathbb E} [{\mathcal T} _{\hat{\beta }}^2] = O({\hat{\beta }}^4). \end{aligned}$$

The terms in bracket in (4.8) are thus $O({\hat{\beta }}^3) + O({\hat{\beta }}^4) + O({\hat{\beta }}^{4(1-\gamma )})$, which is $o({\hat{\beta }}^2)$ provided we choose $4(1-\gamma ) > 2$, i.e. $\gamma < \frac{1}{2}$. This concludes the proof of (4.5) and Theorem 1.5. $\square$

## Appendix A: Random closed subsets of $\varvec{{\mathbb {R}}}$

In this section, we give a self-contained account of the theoretical background needed to study random closed sets of ${\mathbb {R}}$.

### A.1 Closed subsets of ${\mathbb {R}}$

We denote by ${\mathcal C}$ the class of all closed subsets of ${\mathbb {R}}$ (including the empty set):

4.9 $$\begin{aligned} {\mathcal C} := \{C \subseteq {\mathbb {R}}: \ C \text { is closed} \}. \end{aligned}$$

We equip the set ${\mathcal C}$ with the so-called Fell–Matheron topology, built as follows.

We first compactify ${\mathbb {R}}$ by defining $\overline{{\mathbb {R}}}:= {\mathbb {R}}\cup \{\pm \infty \}$, equipped with the metric

4.10 $$\begin{aligned} d(x, y) = |\arctan (y) - \arctan (x)| \qquad \text{ for } \text{ all } x,y \in \overline{{\mathbb {R}}}. \end{aligned}$$

The Hausdorff distance of two compact non-empty subsets $K, K' \subseteq \overline{{\mathbb {R}}}$ is defined by

4.11 $$\begin{aligned} d_\mathrm{H}(K, K') := \max \Big \{ \sup _{x\in K} d(x, K') \, , \, \sup _{x'\in K'} d(x', K) \Big \} , \end{aligned}$$

where $d(a, B) :=\inf _{b\in B}d(a,b)$. (Note that $d_H(K, K')\le \varepsilon$ if and only if for each $x\in K$ there is $x'\in K'$ with $d(x, x')\le \varepsilon$, and vice versa switching the roles of $K$ and $K'$.)

Coming back to ${\mathcal C}$, one can identify a closed subset $C \subseteq {\mathbb {R}}$ with the compact non-empty subset $C \cup \{\pm \infty \} \subseteq \overline{{\mathbb {R}}}$. This allows to define a metric $d_\mathrm{FM}$ on ${\mathcal C}$:

4.12 $$\begin{aligned} d_\mathrm{FM}(C, C') := d_\mathrm{H}(C \cup \{\pm \infty \}, C' \cup \{\pm \infty \}), \qquad C, C' \in {\mathcal C} . \end{aligned}$$

The topology induced by the distance $d_\mathrm{FM}$ on ${\mathcal C}$ is called the Fell–Matheron topology [24, Prop. 1-4-4 and Remark on p.14]. Since the metric space $({\mathcal C} , d_\mathrm{FM})$ is *compact* (hence separable and complete)  [24, Th. 1-2-1], it follows that ${\mathcal C}$ is a *Polish space*.

#### *Remark A.1*

By (4.11)–(4.12), $C_n \rightarrow C$ in ${\mathcal C}$ if and only if the following conditions hold:

- for every open set $G \subseteq {\mathbb {R}}$ with $G \cap C \ne \emptyset$, one has $G \cap C_n \ne \emptyset$ for large $n$;
- for every compact set $K \subseteq {\mathbb {R}}$ with $K \cap C = \emptyset$, one has $K \cap C_n = \emptyset$ for large $n$.

We also observe that the Fell–Matheron topology on closed subsets can be studied for more general topological space, together with the topology induced by the Hausdorff metric (4.11) on compact non-empty subsets (called myope topology): for more details, we refer to [24], [25, Appendixes B and C] and [30, Appendix B].

### A.2 Finite-dimensional distributions

The space ${\mathcal C}$ is naturally equipped with the Borel $\sigma$-algebra ${\mathcal B} ({\mathcal C} )$ generated by the open sets. By *random closed subset of* ${\mathbb {R}}$ we mean any ${\mathcal C}$-valued random variable $X$. We are going to characterize the law of $X$, which is a probability measure on ${\mathcal C}$, in terms of suitable *finite-dimensional distributions*, which provide useful criteria for convergence in distribution.

To every element $C\in {\mathcal C}$ we associate two non-decreasing and right-continuous functions $t \mapsto \mathtt g_t(C)$ and $t \mapsto \mathtt d_t(C)$, defined for $t \in {\mathbb {R}}$ with values in $\overline{{\mathbb {R}}}$ as follows:

4.13 $$\begin{aligned} \mathtt g_t(C) := \sup \{x: \ x\in C, \ x \le t\}, \qquad \mathtt d_t(C) := \inf \{x: \ x\in C, \ x > t\} \end{aligned}$$

(where $\sup \emptyset := -\infty$ and $\inf \emptyset := +\infty$). Note that *either function determines the set* $C$, because $t \in C$ if and only if $\mathtt g_t(C) = t$ if and only if $\mathtt d_{t-}(C) = t$. It is therefore natural to describe a random closed set $X$ in terms of the random functions $t \mapsto \mathtt g_t(X)$ and $t \mapsto \mathtt d_t(X)$.

For convenience, we state results for both $\mathtt g$ and $\mathtt d$, even if one could focus only on one of the two. We start with some basic properties of the maps $\mathtt g_t(\cdot )$ and $\mathtt d_t(\cdot )$.

#### **Lemma A.2**

For every $t\in {\mathbb {R}}$, consider $\mathtt g_t(\cdot )$ and $\mathtt d_t(\cdot )$ as maps from ${\mathcal C}$ to $\overline{{\mathbb {R}}}$.

(i) These maps are measurable with respect to the Borel $\sigma$-algebra ${\mathcal B} ({\mathcal C} )$, and they generate it as the index $t$ ranges in a dense set ${\mathcal T} \subseteq {\mathbb {R}}$, i.e. ${\mathcal B} ({\mathcal C} ) = \sigma ((\mathtt g_t)_{t\in {\mathcal T} }) = \sigma ((\mathtt d_t)_{t\in {\mathcal T} })$.
(ii) These maps are not continuous on ${\mathcal C}$. In fact, the map $\mathtt g_t(\cdot )$ is continuous at $C \in {\mathcal C}$ if and only if the function $\mathtt g_\cdot (C)$ is continuous at $t$. The same holds for $\mathtt d_t(\cdot )$.

Given a ${\mathcal C}$-valued random variable $X$, we call $\mathtt g$-*finite-dimensional distributions* ($\mathtt g$-*f.d.d.*) *of* $X$ the laws of the random vectors $(\mathtt g_{t_1}(X), \ldots , \mathtt g_{t_k}(X))$, for $k\in {\mathbb {N}}$ and $t_1, \ldots , t_k \in {\mathbb {R}}$. Analogously, we call $\mathtt d$-f.d.d. the laws of the random vectors $(\mathtt d_{t_1}(X), \ldots , \mathtt d_{t_k}(X))$. We simply write f.d.d. to mean either $\mathtt g$-f.d.d. or $\mathtt d$-f.d.d., or both, when no confusion arises.

Since $X$ is determined by the functions $t \mapsto \mathtt g_t(X)$ and $t \mapsto \mathtt d_t(X)$, it is not surprising that the law of $X$ on ${\mathcal C}$ is uniquely determined by its f.d.d., and that criteria for convergence in distribution $X_n \Rightarrow X$ of ${\mathcal C}$-valued random variables can be given in terms of f.d.d.. Some care is needed, however, because the maps $\mathtt g_t(\cdot )$ and $\mathtt d_t(\cdot )$ are not continuous on ${\mathcal C}$. For this reason, given a ${\mathcal C}$-valued random variable $X$, we denote by ${\mathcal I} _\mathtt g(X)$ the subset of those $t \in {\mathbb {R}}$ for which the function $s \mapsto \mathtt g_s(X)$ is continuous at $s=t$ with probability one:

4.14 $$\begin{aligned} {\mathcal I} _\mathtt g(X) := \big \{t \in {\mathbb {R}}: \ \mathrm P\big (\mathtt g_{t-}(X) = \mathtt g_{t}(X) \big ) = 1 \big \}. \end{aligned}$$

One defines ${\mathcal I} _\mathtt d(X)$ analogously. We then have the following result.

#### **Proposition A.3**

(Characterization and convergence via f.d.d.) Let $(X_n)_{n\in {\mathbb {N}}}$, $X$ be ${\mathcal C}$-valued random variables.

(i) The set ${\mathcal I} _\mathtt g(X)$ is cocountable, i.e. ${\mathbb {R}}{\setminus }{\mathcal I} _\mathtt g(X)$ is at most countable.
(ii) The law of $X$ is determined by its $\mathtt g$-f.d.d. with indices $t_1,\ldots , t_k$ in a dense set ${\mathcal T} \subseteq {\mathbb {R}}$.
(iii) Assume that $X_n \Rightarrow X$. Then the $\mathtt g$-f.d.d. of $X_n$ with indices in the cocountable set ${\mathcal I} _\mathtt g(X)$ converge weakly to the $\mathtt g$-f.d.d. of $X$: for all $k\in {\mathbb {N}}$ and $t_1, \ldots , t_k \in {\mathcal I} _\mathtt g(X)$,
  $$\begin{aligned} (\mathtt g_{t_1}(X_n), \ldots , \mathtt g_{t_k}(X_n)) \Rightarrow (\mathtt g_{t_1}(X), \ldots , \mathtt g_{t_k}(X)) . \end{aligned}$$
(iv) Assume that the $\mathtt g$-f.d.d. of $X_n$ with indices in a set ${\mathcal T} \subseteq {\mathbb {R}}$ with full Lebesgue measure converge weakly: for $k\in {\mathbb {N}}$, $t_1, \ldots , t_k \in {\mathcal T}$ there are measures $\mu _{t_1, \ldots , t_k}$ on $\overline{{\mathbb {R}}}^k$ such that
  $$\begin{aligned} (\mathtt g_{t_1}(X_n), \ldots , \mathtt g_{t_k}(X_n)) \Rightarrow \mu _{t_1, \ldots , t_k}. \end{aligned}$$
  Then there is a ${\mathcal C}$-valued random variable $X$ such that $X_n \Rightarrow X$. In particular, the $\mathtt g$-f.d.d. of $X$ with indices in the set ${\mathcal T} \cap {\mathcal I} _\mathtt g(X)$ are given by $\mu _{t_1, \ldots , t_k}$.

The same conclusions hold replacing $\mathtt g$ by $\mathtt d$.

#### *Remark A.4*

In Proposition A.3 (iv) it is sufficient that ${\mathcal T}$ has uncountably many points in every non-empty open interval $(a,b) \subseteq {\mathbb {R}}$, as the proof shows. In fact, arguing as in [14, Th. 7.8 in Ch. 3], it is even enough that ${\mathcal T}$ is dense in ${\mathbb {R}}$ (in which case the f.d.d. of $X$ must be recovered from $\mu _{t_1,\ldots ,t_n}$ by a limiting procedure, since ${\mathcal T} \cap {\mathcal I} _\mathtt g(X)$ could be empty).

#### *Remark A.5*

The map $C \mapsto (\mathtt g_t(C))_{t\in {\mathbb {R}}}$ allows one to identify ${\mathcal C}$ with a class of functions ${\mathcal D} _0$ that can be explicitly described:

4.15 $$\begin{aligned}&{\mathcal D} _0:= \{ f: {\mathbb {R}}\rightarrow {\mathbb {R}}\cup \{-\infty \} : \ \, f(t)\le t \hbox { and } f(t+)=f(t), \ \forall \, t\in {\mathbb {R}}; \nonumber \\&\qquad \qquad \text {if } f(t)<u \text { for some } u<t, \text { then } f(u)=f(t) \} . \end{aligned}$$

The functions in ${\mathcal D} _0$ are non-decreasing and right-continuous, hence càdlàg, and it turns out that the Fell–Matheron topology on ${\mathcal C}$ corresponds to the *Skorokhod topology* on ${\mathcal D} _0$. As a matter of fact, given the structure of ${\mathcal D} _0$, convergence $f_n \rightarrow f$ in the Skorokhod topology is equivalent to pointwise convergence $f_n(x) \rightarrow f(x)$ at all continuity points $x$ of $f$.

We do not prove these facts, because we do not use them directly. However, as the reader might have noticed, the key results of this section are translations of analogous results for the Skorokhod topology [6, 14, 21].

### A.3 Restricted finite-dimensional distributions

It turns out that, in order to describe the f.d.d. of a ${\mathcal C}$-valued random variable $X$, say the law of $(\mathtt g_{t_1}(X), \ldots , \mathtt g_{t_k}(X))$, with $-\infty < t_1 < \cdots < t_k < +\infty$, it is sufficient to focus on the event

4.16 $$\begin{aligned} A_{t_1,\ldots , t_k}^X := \{X \cap (t_{1},t_{2}] \ne \emptyset , \ X \cap (t_{2},t_{3}] \ne \emptyset , \ \ldots \ , X \cap (t_{k-1},t_{k}] \ne \emptyset \}.\qquad \end{aligned}$$

We thus define the *restricted* $\mathtt g$-*f.d.d.* of $X$ as the laws of the vectors $(\mathtt g_{t_1}(X), \ldots , \mathtt g_{t_k}(X))$ *restricted on the event* $A_{t_1,\ldots , t_k}^X$. These are sub-probabilities, i.e. measures with total mass $\mathrm P(A_{t_1,\ldots , t_k}^X) \le 1$, and we equip them with the usual topology of weak convergence with respect to bounded and continuous functions. One defines analogously the restricted $\mathtt d$-f.d.d. of $X$. We can then rephrase Proposition A.3 as follows.

#### **Proposition A.6**

(Characterization and convergence via restricted f.d.d.) Let $(X_n)_{n\in {\mathbb {N}}}$, $X$ be ${\mathcal C}$-valued random variables.

(i) The law of $X$ is determined by its restricted $\mathtt g$-f.d.d. with indices in a dense set ${\mathcal T} \subseteq {\mathbb {R}}$.
(ii) Assume that $X_n \Rightarrow X$. Then the restricted $\mathtt g$-f.d.d. of $X_n$ with indices in the cocountable set ${\mathcal I} _\mathtt g(X) \cap {\mathcal I} _\mathtt d(X)$ converge weakly to those of $X$.
(iii) Assume that the restricted $\mathtt g$-f.d.d. of $X_n$ with indices in a set ${\mathcal T} \subseteq {\mathbb {R}}$ with full Lebesgue measure converge weakly to some measures $\mu ^\mathrm{rest}_{t_1,\ldots ,t_k}$ on $\overline{{\mathbb {R}}}^k$. Then there is a ${\mathcal C}$-valued random variable $X$ such that $X_n \Rightarrow X$. In particular, the restricted $\mathtt g$-f.d.d. of $X$ with indices in the set ${\mathcal T} \cap {\mathcal I} _\mathtt g(X) \cap {\mathcal I} _\mathtt d(X)$ are given by $\mu ^\mathrm{rest}_{t_1, \ldots , t_k}$.

The same conclusions hold replacing $\mathtt g$ by $\mathtt d$.

#### *Remark A.7*

Note that $X \cap (s,t] \ne \emptyset$ if and only if $s \le \mathtt d_s(X) \le \mathtt g_t(X) \le t$. Recalling the definition (1.18) of the set ${\mathcal R} ^{(k)}_{t_0, t_1, \ldots , t_k, t_{k+1}}$, the event $A_{t_1,\ldots , t_k}^X$ in (4.16) can be rewritten as

4.17 $$\begin{aligned} A_{t_1,\ldots , t_k}^X = \big \{ (\mathtt g_{t_1}(X), \mathtt d_{t_1}(X), \ldots , \mathtt g_{t_k}(X), \mathtt d_{t_k}(X)) \in {\mathcal R} ^{(k)}_{-\infty , t_1, \ldots , t_k, \infty } \big \}. \end{aligned}$$

Also note that, in case $X \subseteq [a,b]$ a.s., it is enough to specify the restricted f.d.d. with indices $t_i \in [a,b]$, using correspondingly ${\mathcal R} ^{(k)}_{a, t_1, \ldots , t_k, b}$ instead of ${\mathcal R} ^{(k)}_{-\infty , t_1, \ldots , t_k, \infty }$ in (4.17).

### A.4 The $\alpha$-stable regenerative set

For each $\alpha \in (0,1)$ there is a universal random closed set $\varvec{\tau }$ of $[0,\infty )$, called the $\alpha$-*stable regenerative set*. Its probability law $\varvec{\mathrm {P}}^\alpha$ on ${\mathcal C}$ is then characterized by the following restricted f.d.d. densities: for all $k\in {\mathbb {N}}$ and $0 < t_1 < \cdots < t_k < \infty$, setting $y_0 := 0$ and $C_\alpha :=\frac{\alpha \sin (\pi \alpha )}{\pi }$,

4.18 $$\begin{aligned}&\frac{\varvec{\mathrm {P}}^{\alpha } \big (\mathtt g_{t_1}(\varvec{\tau }) \in \mathrm {d}x_1,\, \mathtt d_{t_1}(\varvec{\tau }) \in \mathrm {d}y_1, \, \ldots \,,\, \mathtt g_{t_k}(\varvec{\tau })\in \mathrm {d}x_k,\, \mathtt d_{t_k}(\varvec{\tau }) \in \mathrm {d}y_k\big )}{\mathrm {d}x_1 \, \mathrm {d}y_1 \, \cdots \, \mathrm {d}x_k \, \mathrm {d}y_k} \nonumber \\&\qquad = \, \mathsf {f}_{t_1,\ldots ,t_k}^{\alpha }(x_1,y_1,\ldots ,x_k,y_k) \, := \, \prod _{i=1}^{k} \frac{C_\alpha }{(x_{i} - y_{i-1})^{1-\alpha } \, (y_i-x_i)^{1+\alpha }} \,,\qquad \end{aligned}$$

restricting $(x_1,y_1, \ldots , x_k, y_k)$ in the set ${\mathcal R} ^{(k)}_{0, t_1, \ldots , t_k, \infty } \subseteq {\mathbb {R}}^{2k}$ (4.17)–(1.18).

The $\alpha$-stable regenerative set can be characterized in many ways (e.g., as the zero level set of a Bessel process of dimension $\delta := 2(1-\alpha ) \in (0,2)$ [27], or as the closure of the range of the $\alpha$-stable subordinator [16]). One of the most expressive is to view it as the *universal scaling limit of discrete renewal processes*: for any renewal process $\tau :=(\tau _n)_{n\in {\mathbb {N}}_0}$ on ${\mathbb {N}}_0$ satisfying (1.1), the rescaled random set $\tau /N$ (1.2), viewed as a ${\mathcal C}$-valued random variable, converges in distribution as $N\rightarrow \infty$ toward the $\alpha$-stable regenerative set.

This can be shown using the general theory of *regenerative sets* [16], but it is instructive to prove it directly, as an application of Proposition A.6 (which shows, as a by-product, that the restricted f.d.d. (4.18) define indeed a probability law on ${\mathcal C}$). We spell this out in the *conditioned case*, which is more directly linked to our main results, but the proof for the unconditioned case is analogous (and actually simpler).

#### **Proposition A.8**

(Characterization and universality of $\alpha$-stable regenerative set)

Let $\tau :=(\tau _n)_{n\in {\mathbb {N}}_0}$ be a renewal process on ${\mathbb {N}}_0$ satisfying (1.1), for some $\alpha \in (0,1)$. For fixed $T>0$, the rescaled random set $\tau /N \cap [0,T]$ (1.2), conditioned on $\lfloor TN\rfloor \in \tau$, converges in distribution as $N\rightarrow \infty$ to the probability law $\varvec{\mathrm {P}}^{\alpha ;\mathrm c}_T$ of the $\alpha$-stable regenerative set $\varvec{\tau }\cap [0,T]$ conditioned on $T\in \varvec{\tau }$ (1.13). This law is characterized by the following restricted f.d.d.: for all $k\in {\mathbb {N}}$ and $0<t_1<\cdots <t_k< T$, and for $(x_1,y_1, \ldots , x_k, y_k)$ in the set ${\mathcal R} ^{(k)}_{0, t_1, \ldots , t_k, T} \subseteq {\mathbb {R}}^{2k}$ (4.17) and (1.18),

4.19 $$\begin{aligned}&\varvec{\mathrm {P}}^{\alpha ;\mathrm c}_T\big (\mathtt g_{t_1}(\varvec{\tau }) \in \mathrm {d}x_1,\, \mathtt d_{t_1}(\varvec{\tau }) \in \mathrm {d}y_1, \ldots , \mathtt g_{t_k}(\varvec{\tau })\in \mathrm {d}x_k,\, \mathtt d_{t_k}(\varvec{\tau }) \in \mathrm {d}y_k\big ) \nonumber \\&\qquad = \ \mathsf {f}_{T;t_1,\ldots ,t_k}^{\alpha ;\mathrm c}(x_1,y_1,\ldots ,x_k,y_k) \,:=\, \mathsf {f}_{t_1,\ldots ,t_k}^{\alpha }(x_1,y_1,\ldots ,x_k,y_k) \, \frac{T^{1-\alpha }}{(T-y_k)^{1-\alpha }} \,,\nonumber \\ \end{aligned}$$

where $\mathsf {f}_{t_1,\ldots ,t_k}^{\alpha }(\cdot )$ are the f.d.d. densities of the $\alpha$-stable regenerative set (4.18).

### Proofs

We now give the proofs of the results stated in the previous subsections.

#### *Proof of Lemma A.2*

We start proving part (ii). Fix $C \in {\mathcal C}$ and $t \in {\mathbb {R}}$ and recall Remark A.1. If the function $\mathtt g_\cdot (C)$ is *not* continuous at $t$, i.e. $\mathtt g_{t-}(C) < \mathtt g_t(C)$, defining $C_n := C + \frac{1}{n}$ (i.e. translating $C$ to the right by $\frac{1}{n}$) one has $C_n \rightarrow C$ as $n\rightarrow \infty$, but $\mathtt g_t(C_n) = \mathtt g_{t-\frac{1}{n}}(C) + \frac{1}{n} \rightarrow \mathtt g_{t-}(C) \ne \mathtt g_t(C)$. Thus $\mathtt g_t(\cdot )$ is *not* continuous at $C$.

Assume now that $\mathtt g_\cdot (C)$ is continuous at $t$. We set $s := \mathtt g_t(C)$ and distinguish two cases.

- If $s < t$, then $t \not \in C$ by (4.13). Assume for simplicity that $s > -\infty$ (the case $s = -\infty$ is analogous). For $\varepsilon > 0$ small one has $(s-\varepsilon , s+\varepsilon ) \cap C \ne \emptyset$, while $[s+\varepsilon , t] \cap C = \emptyset$. If $C_n \rightarrow C$, then $(s-\varepsilon , s+\varepsilon ) \cap C_n \ne \emptyset$ and $[s+\varepsilon , t] \cap C_n = \emptyset$ for large $n$, hence $\mathtt g_t(C_n) \in (s-\varepsilon , s+\varepsilon )$. This shows that $\mathtt g_t(C_n) \rightarrow \mathtt g_t(C)$, i.e., $\mathtt g_t(\cdot )$ is continuous at $C$.
- If $s = t$, since $\mathtt g_{t-}(C) = \mathtt g_t(C) = t$, for every $\varepsilon > 0$ one has $(t-\varepsilon , t) \cap C \ne \emptyset$. If $C_n \rightarrow C$, then $(t-\varepsilon , t) \cap C_n \ne \emptyset$ for large $n$, hence $\mathtt g_t(C_n) \in (t-\varepsilon , t)$. This shows that $\mathtt g_t(C_n) \rightarrow \mathtt g_t(C)$, that is, $\mathtt g_t(\cdot )$ is continuous at $C$.

Thus $\mathtt g_t(\cdot )$ is continuous at $C \in {\mathcal C}$ if and only if $\mathtt g_\cdot (C)$ is continuous at $t$, proving part (ii).

We now turn to part (i). Defining $G_{t;m,\varepsilon }(C) := \frac{1}{\varepsilon } \int _t^{t+\varepsilon } \max \{\mathtt g_s(C), -m\} \, \mathrm {d}s$, we can write $\mathtt g_t(C) = \lim _{m \rightarrow \infty } \lim _{n\rightarrow \infty } G_{t;m, 1/n}(C)$ for all $t\in {\mathbb {R}}$ and $C\in {\mathcal C}$. The measurability of $\mathtt g_t(\cdot )$ will follow if we show that $G_{t;m, \varepsilon }(\cdot )$ is continuous, and hence measurable. If $C_n \rightarrow C$ in ${\mathcal C}$, we know that $\mathtt g_s(C_n) \rightarrow \mathtt g_s(C)$ at continuity points $s$ of the non-decreasing function $\mathtt g_\cdot (C)$, hence for Lebesgue a.e. $s\in {\mathbb {R}}$. Since $\mathtt g_s(C) \le s$, dominated convergence yields $G_{t;m, \varepsilon }(C_n) \rightarrow G_{t;m, \varepsilon }(C)$ as $n\rightarrow \infty$, i.e. the function $G_{t;m, \varepsilon }(\cdot )$ is continuous on ${\mathcal C}$.

Finally, setting ${\mathcal B} ' := \sigma ((\mathtt g_t)_{t\in {\mathcal T} })$, where ${\mathcal T} \subseteq {\mathbb {R}}$ is a fixed dense set, the measurability of the maps $\mathtt g_t(\cdot )$ yields ${\mathcal B} ' \subseteq {\mathcal B} ({\mathcal C} )$. If we exhibit measurable maps $\psi _n: ({\mathcal C} , {\mathcal B} ') \rightarrow ({\mathcal C} , {\mathcal B} ({\mathcal C} ))$ such that $C = \lim _{n\rightarrow \infty } \psi _n(C)$ in ${\mathcal C}$, for all $C\in {\mathcal C}$, it follows that the identity map $\psi (C) := C$ is measurable from $({\mathcal C} , {\mathcal B} ')$ to $({\mathcal C} , {\mathcal B} ({\mathcal C} ))$, as the pointwise limit of $\psi _n$, hence ${\mathcal B} ({\mathcal C} ) \subseteq {\mathcal B} '$.

Extracting a *countable* dense set $\{t_i\}_{i\in {\mathbb {N}}} \subseteq {\mathcal T}$, we define $\psi _n(C) := \{\mathtt g_{t_1}(C), \ldots , \mathtt g_{t_n}(C)\} \cap {\mathbb {R}}$, so that $\psi _n(C)$ is a finite subset of ${\mathbb {R}}$ and $\psi _n(C) \rightarrow C$ in ${\mathcal C}$. Since $\mathtt g_t(\cdot )$ is a measurable map from $({\mathcal C} , {\mathcal B} ')$ to $\overline{{\mathbb {R}}}$ and $(x_1, \ldots , x_n) \mapsto \{x_1, \ldots , x_k\} \cap {\mathbb {R}}$ is a continuous, hence measurable, map from ${\overline{{\mathbb {R}}}}^k$ to $({\mathcal C} , {\mathcal B} ({\mathcal C} ))$, it follows that $\psi _n: ({\mathcal C} , {\mathcal B} ') \rightarrow ({\mathcal C} , {\mathcal B} ({\mathcal C} ))$ is measurable.$\square$

#### *Proof of Proposition A.3*

Since the path $t \mapsto \mathtt g_t(X)$ is increasing and right-continuous, its discontinuity points $t$, at which $\mathtt g_t(X) \ne \mathtt g_{t-}(X)$, are at most countably many, a.s.. The corresponding fact that $\mathrm P(\mathtt g_t(X) \ne \mathtt g_{t-}(X)) > 0$ is possible for at most countably many $t$ follows by a classilcal argument, see e.g. [6, Section 13]. This proves part (i).

The proof of part (ii) is an easy consequence of Lemma A.2. A generator for the Borel $\sigma$-algebra ${\mathcal B} (C)$ is given by sets of the form $\{C\in {\mathcal C} : \ \mathtt g_{t_1}(C) \in A_1, \ldots , \mathtt g_{t_k}(C) \in A_k\}$, for $k\in {\mathbb {N}}$, $t_1,\ldots , t_k \in {\mathcal T}$ and $A_1, \ldots , A_k$ Borel subsets of ${\mathcal B}$. Note that such sets are a $\pi$-system, i.e. they are closed under finite intersections. It is then a standard result that any probability on $({\mathcal C} , {\mathcal B} (C))$ — in particular, the distribution of any ${\mathcal C}$-valued random variable $X$ — is characterized by its values on such sets, i.e. by its finite-dimensional distributions.

We now turn to part (iii). If $X_n \Rightarrow X$ on ${\mathcal C}$, by Skorokhod’s Representation Theorem [6, Th.6.7] we can couple $X_n$ and $X$ so that a.s. $X_n \rightarrow X$ in ${\mathcal C}$. If $t_i, \ldots , t_k \in {\mathcal I} _\mathtt g(X)$, the maps $\mathtt g_{t_i}(\cdot )$ are a.s. continuous at $X$, by Lemma A.2 (ii), hence one has the a.s. convergence $(\mathtt g_{t_1}(X_n), \ldots , \mathtt g_{t_k}(X_n)) \rightarrow (\mathtt g_{t_1}(X), \ldots , \mathtt g_{t_k}(X))$, which implies weak convergence of the f.d.d..

We finally prove part (iv). Since ${\mathcal C}$ is a compact Polish space, every sequence $(X_n)_{n\in {\mathbb {N}}}$ of ${\mathcal C}$-valued random variables is tight, and hence relatively compact for the topology of convergence in distribution [6, Th.2.7]. We can then extract a subsequence $X_{n_k}$ converging in distribution to some ${\mathcal C}$-valued random variable $X$. To show that the whole sequence $X_n$ converges to $X$, by [6, Th.2.6] it is enough to show that for any other converging subsequence $X_{n'_k} \Rightarrow X'$, the random variables $X$ and $X'$ have the same distribution.

By assumption, the f.d.d. of $X_n$ with indices $t_1,\ldots , t_k$ in a set ${\mathcal T} \subseteq {\mathbb {R}}$ with full Lebesgue measure converge to $\mu _{t_1,\ldots , t_k}$. Since $X_{n_k} \Rightarrow X$, the f.d.d. of $X$ are given by $\mu _{t_1,\ldots , t_k}$ for indices in ${\mathcal T} \cap {\mathcal I} _\mathtt g(X)$, by part (iii); analogously, the f.d.d. of $X'$ are given by $\mu _{t_1,\ldots , t_k}$ for indices in ${\mathcal T} \cap {\mathcal I} _\mathtt g(X')$. Thus $X$ and $X'$ have the same f.d.d. with indices in ${\mathcal T} \cap {\mathcal I} _\mathtt g(X) \cap {\mathcal I} _\mathtt g(X')$. Since this set is dense, $X$ and $X'$ have the same distribution, by Proposition A.3 (ii).$\square$

#### *Proof of Proposition A.6*

We start proving part (i). Fix a ${\mathcal C}$-valued random variable $X$ and let $\mu _{t_1, \ldots , t_k}(\mathrm {d}x_1, \ldots , \mathrm {d}x_k)$ be the $\mathtt g$-f.d.d. of $X$, i.e. the law on $\overline{{\mathbb {R}}}^k$ of $(\mathtt g_{t_1}(X), \ldots , \mathtt g_{t_k}(X))$. Analogously, let $\mu ^\mathrm{rest}_{t_1, \ldots , t_k}(\mathrm {d}x_1, \ldots , \mathrm {d}x_k)$ be the restricted $\mathtt g$-f.d.d. of $X$ (4.16)–(4.17). Since $X \cap (s,t] \ne \emptyset$ if and only if $\mathtt g_t(X) \in (s,t]$, the restricted f.d.d. $\mu ^\mathrm{rest}_{t_1, \ldots , t_k}$ is just the f.d.d. $\mu _{t_1, \cdots , t_k}$ restricted on the subset $[-\infty ,t_1] \times (t_1,t_2] \times \cdots \times (t_{k-1},t_k]$:

4.20 $$\begin{aligned} \mu ^\mathrm{rest}_{t_1, \ldots , t_k}(\mathrm {d}x_1, \ldots , \mathrm {d}x_k) = \mu _{t_1, \ldots , t_k}(\mathrm {d}x_1, \ldots , \mathrm {d}x_k) \prod _{i=2}^k 1\!\!1_{\{x_i \in (t_{i-1},t_i]\}}. \end{aligned}$$

To prove that the f.d.d. can be recovered from the restricted f.d.d., we show that $\mu _{t_1, \ldots , t_k}(\cdot )$ can be written as a mixture of $\mu ^\mathrm{rest}_{I}(\cdot )$, as $I$ ranges in the subsets of $\{t_1, \ldots , t_k\}$. For $k=1$ there is nothing to prove, since $\mu _{t_1} = \mu ^\mathrm{rest}_{t_1}$, so we assume $k\ge 2$ henceforth. We associate to $X$ the (possibly empty) subset $\fancyscript{B}(X) \subseteq \{1, \ldots , k-1\}$ defined by

$$\begin{aligned} \fancyscript{B}(X) \,:=\, \big \{ j \in \{1, \ldots , k-1\}: \ X \cap (t_{j}, t_{j+1}] \ne \emptyset \big \}. \end{aligned}$$

Performing a decomposition over the possible values of $\fancyscript{B}(X)$, we can write

4.21 $$\begin{aligned} \mu _{t_1, \ldots , t_k}(\mathrm {d}x_1, \ldots , \mathrm {d}x_k) \,= \!\!\! \sum _{B \subseteq \{2,\ldots , k\}} \!\!\! \mathrm P(\mathtt g_{t_1}(X) \in \mathrm {d}x_1,\, \ldots ,\, \mathtt g_{t_k}(X) \in \mathrm {d}x_k , \, \fancyscript{B}(X) = B). \end{aligned}$$

It remains to express each term in the right hand side in terms of restricted f.d.d..

We first consider the case $B = \emptyset$. On the event $\{\fancyscript{B}(X) = \emptyset \}=\{X \cap (t_1, t_k] = \emptyset \}$ we have $\mathtt g_{t_1}(X) = \cdots = \mathtt g_{t_k}(X) \le t_1$. Recalling that $\mu _{t} = \mu ^\mathrm{rest}_{t}$, we can write

$$\begin{aligned}&\mathrm P(\mathtt g_{t_1}(X) \in \mathrm {d}x_1,\, \ldots ,\, \mathtt g_{t_k}(X) \in \mathrm {d}x_k , \, \fancyscript{B}(X) = \emptyset )\\&\quad =\ \mathrm P(\mathtt g_{t_k}(X) \in \mathrm {d}x_k) \ 1\!\!1_{\{x_k \le t_1\}} \, \prod _{i=1}^{k-1} \delta _{x_k}(\mathrm {d}x_i) \ = \ \mu ^\mathrm{rest}_{t_k}(\mathrm {d}x_k) \ 1\!\!1_{\{x_k \le t_1\}} \, \prod _{i=1}^{k-1} \delta _{x_k}(\mathrm {d}x_i) , \end{aligned}$$

and this expression depends only on the restricted f.d.d..

If $B \ne \emptyset$, we can write $B = \{j_1, \ldots , j_\ell \}$ with $1 \le \ell \le k-1$ and $1 \le j_1 < \ldots < j_\ell \le k-1$. Let us also set $j_0 := 0$ and $j_{\ell + 1} := k$. On the event $\{\fancyscript{B} = B\}$, we have $\mathtt g_{t_{j_{n-1}+1}}(X) = \mathtt g_{t_{j_{n-1}+2}}(X) = \ldots = \mathtt g_{t_{j_n}}(X) \in (t_{j_{n-1}}, t_{j_{n-1}+1}]$, for every $n=1, \ldots , \ell + 1$, therefore

4.22 $$\begin{aligned}&\mathrm P(\mathtt g_{t_1}(X) \in \mathrm {d}x_1,\, \ldots ,\, \mathtt g_{t_k}(X) \in \mathrm {d}x_k , \, \fancyscript{B}(X) = \{j_1, \ldots , j_\ell \}) \nonumber \\&\qquad = \mathrm P\Bigg ( \bigcap _{n=1}^{\ell +1} \big \{ \mathtt g_{t_{j_{n}}}(X) \in \mathrm {d}x_{j_{n}} \big \} \Bigg ) \left\{ \prod _{n=1}^{\ell +1} 1\!\!1_{\{x_{j_{n}} \!\in \! (t_{j_{n-1}}, t_{j_{n-1}+1}]\}} \Bigg (\prod _{m=j_{n-1}+1}^{j_{n} - 1} \!\delta _{x_{j_{n}}}( \mathrm {d}x_{m}) \Bigg ) \right\} ,\nonumber \\ \end{aligned}$$

where we set $(t_0, \,\cdot \,] := [-\infty , \,\cdot \,]$ and the product over $m$ equals one when $j_{n} - j_{n-1} = 1$. The first term in the right hand side of (4.22) is the f.d.d. $\mu _{t_{j_1}, \ldots , t_{j_{\ell +1}}}(\mathrm {d}x_{j_1}, \ldots , \mathrm {d}x_{j_{\ell +1}})$. However, by (4.20), *this coincides with the restricted f.d.d.* $\mu ^\mathrm{rest}_{t_{j_1}, \ldots , t_{j_{\ell +1}}}(\mathrm {d}x_{j_1}, \ldots , \mathrm {d}x_{j_{\ell +1}})$, because each variable $x_{j_n}$ is restricted on $(t_{j_{n-1}}, t_{j_{n-1}+1}] \subseteq (t_{j_{n-1}}, t_{j_{n}}]$. Part (i) is thus proved.

For part (ii), we proceed as in the proof of Proposition A.3 (iii). If $X_n \Rightarrow X$ on ${\mathcal C}$, we couple $X_n$ and $X$ so that a.s. $X_n \rightarrow X$ in ${\mathcal C}$, by Skorokhod’s Representation Theorem. On the event that $\mathtt g_\cdot (X)$ is continuous at $t$, if $X \cap (s,t] \ne \emptyset$ then also $X \cap (s,t) \ne \emptyset$, which implies $X_n \cap (s,t) \ne \emptyset$ for large $n$ (Remark A.1). Analogously, on the event that $\mathtt d_\cdot (X)$ is continuous at $s$, if $X \cap (s,t] = \emptyset$ then also $X \cap [s,t] = \emptyset$, which implies $X_n \cap [s,t] = \emptyset$ for large $n$. Therefore, if $t_1, \ldots , t_k \in {\mathcal I} _\mathtt g(X) \cap {\mathcal I} _\mathtt d(X)$ and $f:\overline{{\mathbb {R}}}^k \rightarrow {\mathbb {R}}$ is bounded and continuous,

$$\begin{aligned}&f\big ( \mathtt g_{t_1}(X_n), \ldots , \mathtt g_{t_k}(X_n) \big ) 1\!\!1_{\{X_n \cap (t_{i-1},t_{i}] \ne \emptyset , \ \forall i = 2, \ldots , k\} } \\&\qquad \qquad \qquad \qquad \xrightarrow [\ n\rightarrow \infty \ ]{\text {a.s.}} f\big ( \mathtt g_{t_1}(X), \ldots , \mathtt g_{t_k}(X) \big ) 1\!\!1_{\{X \cap (t_{i-1},t_{i}] \ne \emptyset , \ \forall i = 2, \ldots , k\} } . \end{aligned}$$

Taking expectations of both sides, dominated convergence shows that the restricted f.d.d. of $X_n$ with indices in ${\mathcal I} _\mathtt g(X) \cap {\mathcal I} _\mathtt d(X)$ converge weakly toward the restricted f.d.d. of $X$.

Finally, the proof of part (iii) is analogous to that of Proposition A.3 (iv). Any sequence $X_n$ of ${\mathcal C}$-valued random variable is tight, hence it suffices to show that if $X_{n_k}$, $X_{n'_k}$ are subsequences converging in distribtion to $X$, $X'$ respectively, then $X$ and $X'$ have the same law. Since the restricted $\mathtt g$-f.d.d. of $X_n$ with indices in ${\mathcal T}$ converge, $X$ and $X'$ have the same restricted $\mathtt g$-f.d.d. with indexes in the dense set ${\mathcal T} \cap {\mathcal I} _\mathtt g(X) \cap {\mathcal I} _\mathtt d(X) \cap {\mathcal I} _\mathtt g(X') \cap {\mathcal I} _\mathtt d(X')$, by part (ii). It follows by part (i) that $X$ and $X'$ have the same law.$\square$

#### *Proof of Proposition A.8*

By Proposition A.6 (iii), it is enough to prove the convergence of restricted f.d.d.: for all $k\in {\mathbb {N}}$, $0 < t_1 < \ldots < t_k < T$ and for every bounded and continuous function $F: {\mathbb {R}}^{2k} \rightarrow {\mathbb {R}}$, recalling (4.16)–(4.17) and (3.3), we show that the integral

4.23 $$\begin{aligned} I_N := \mathrm E_N^\mathrm{c} \Big [ F\big (\mathtt g_{t_1}(\tau /N), \, \mathtt d_{t_1}(\tau /N), \ldots , \mathtt g_{t_k}(\tau /N), \mathtt d_{t_k}(\tau /N)\big ) 1\!\!1_{A_{t_1,\ldots , t_k}^{\tau /N}} \Big ] \end{aligned}$$

converges as $N\rightarrow \infty$ to the integral of $F$ with respect to the density $\mathsf {f}_{T;t_1,\ldots ,t_k}^{\alpha ;\mathrm c}$ in (4.19), i.e.

4.24 $$\begin{aligned}&I := \mathop {\mathop {\idotsint }\limits _{0<x_1<t_1<y_1<x_2<t_2}}\limits _ {<\cdots <x_k<t_k<y_k<T} F(x_1,y_1,\ldots , x_k, y_k) \nonumber \\&\qquad \qquad \times \Bigg [ \prod _{i=1}^{k} \frac{C_\alpha }{(x_{i} - y_{i-1})^{1-\alpha } (y_i-x_i)^{1+\alpha }} \Bigg ] \frac{T^{1-\alpha }}{(T-y_k)^{1-\alpha }} \, \mathrm {d}x \, \mathrm {d}y \,, \end{aligned}$$

where $y_0 := 0$ and $\mathrm {d}x \, \mathrm {d}y$ is a shorthand for $\mathrm {d}x_1 \mathrm {d}y_1 \cdots \mathrm {d}x_k \mathrm {d}y_k$.

Recall that $u(i) := \mathrm P(i\in \tau )$ and $K(i):=\mathrm P(\tau _1=i)$. A renewal decomposition yields

4.25 $$\begin{aligned} I_N&= \frac{1}{N^{2k}} \sum _{0 \le a_1 \le Nt_1} \, \sum _{N t_1 < b_1 \le a_2 \le Nt_2} \cdots \sum _{N t_{k-1} < b_{k-1} \le a_k \le Nt_k} \sum _{N t_k < b_k \le NT}\nonumber \\&\times \, F\Big (\frac{a_1}{N}, \frac{b_1}{N}, \ldots , \frac{a_k}{N}, \frac{b_k}{N}\Big ) \nonumber \\&\times \, \left[ \prod _{i=1}^{k} N^2 \, u(a_i - b_{i-1}) K(b_i - a_i) \right] \frac{u(\lfloor NT\rfloor -b_k)}{u(\lfloor NT\rfloor )} , \end{aligned}$$

where we write the factor $\frac{1}{N^{2k}}$ (which cancels with the product of the $N^2$ inside the square brackets) to make $I_N$ appear as a Riemann sum. Setting $x_i = a_i/N$, $y_i = b_i/N$ for $1\le i\le k$, the summand converges pointwise to the integrand in (4.24), since by (1.1) and (2.10)

$$\begin{aligned} \forall \, z > 0: \quad K(\lfloor Nz \rfloor )\sim \frac{L(Nz)}{(Nz) ^{1+\alpha }}, \quad u(\lfloor Nz\rfloor )\sim \frac{C_\alpha }{L(Nz) (Nz)^{1-\alpha }}, \quad \text {as } N\rightarrow \infty . \end{aligned}$$

To conclude that $I_N$ converges to the integral $I$ in (4.24), we provide a suitable domination. By Potter bounds [8, Theorem1.5.6], for every $\varepsilon > 0$ there is a constant $D_\varepsilon$ such that $L(m)/L(\ell ) \le D_\varepsilon \max \{ (m/\ell )^{\varepsilon }, (\ell /m)^{\varepsilon }\}$ for all $\ell ,m\in {\mathbb {N}}$. Since $(y_{i}-x_{i}) \le T$, $(x_{i} - y_{i-1}) \le T$ and $\max \{\alpha ,\beta \} \le \alpha \beta$ for $\alpha ,\beta \ge 1$, we can write

$$\begin{aligned} \frac{L(b_i-a_i)}{L(a_i-b_{i-1})}&\le D_\varepsilon \max \bigg \{ \frac{ (y_{i}-x_{i})^{\varepsilon }}{(x_{i} - y_{i-1})^{\varepsilon }}, \frac{(x_{i} - y_{i-1})^{\varepsilon }}{ (y_{i}-x_{i})^{\varepsilon }} \bigg \} \le \frac{D_\varepsilon \, T^{2\varepsilon }}{(x_{i} - y_{i-1})^{\varepsilon } (y_{i}-x_{i})^{\varepsilon }} ,\\ \frac{L(\lfloor NT\rfloor )}{L(\lfloor NT\rfloor -b_k)}&\le D_\varepsilon \frac{ T^{\varepsilon }}{(T - y_{k})^{\varepsilon }} . \end{aligned}$$

It follows that, for every $\varepsilon > 0$, the summand in $I_N$ is bounded uniformly in $N$ by

4.26 $$\begin{aligned} C(T,\varepsilon ) \left[ \prod _{i=1}^{k} \frac{1}{(x_{i} - y_{i-1})^{1-\alpha +\varepsilon } (y_i-x_i)^{1+\alpha +\varepsilon }} \right] \frac{1}{(T-y_k)^{1-\alpha +\varepsilon }} . \end{aligned}$$

It remains to show that, if we choose $\varepsilon > 0$ sufficiently small, this function has finite integral over the domain of integration in (4.24). Let us set $\eta := \min _{i=1,\ldots ,k+1} (t_{i} - t_{i-1})$ and

$$\begin{aligned} \delta _i := x_i - y_{i-1} \quad \text {for } i=1,\ldots ,k+1, \qquad \delta '_i := y_i - x_i \quad \text {for } i=1,\ldots ,k, \end{aligned}$$

where $t_0 = y_0 := 0$ and $x_{k+1} = t_{k+1} := T$. Each of the quantities $\delta _i$, $\delta '_i$ can be smaller or larger than $\eta /3$, and we split the integral of (4.26) accordingly, as a sum of $2^{2k+1}$ terms. Note that if $\delta _i < \eta /3$, either $\delta '_{i-1}$ or $\delta '_i$ must exceed $\eta /3$ (because $x_{i-1} < t_{i-1} < t_i < y_i$ and $t_{i} - t_{i-1} \ge \eta$). Whenever any $\delta _i$ or $\delta '_i$ exceeds $\eta /3$, we replace them by $\eta /3$, getting an upper bound. This yields a factorization into a product of just four kind of basic integrals, i.e.

$$\begin{aligned} \mathop {\mathop {\idotsint }\limits _{ y_{i-1} < x_i < t_i < y_i < x_{i+1}}}\limits _{x_i- y_{i-1} < \frac{\eta }{3},\ y_i-x_i < \frac{\eta }{3},\ x_{i+1}-y_i < \frac{\eta }{3}} \!\!\!\!\!\!\!\!\!\!\!\!\!\! \frac{\mathrm {d}y_{i-1}}{(x_i - y_{i-1})^{1-\alpha +\varepsilon }} \frac{\mathrm {d}x_{i} \, \mathrm {d}y_i}{(y_i - x_{i})^{1+\alpha +\varepsilon }} \frac{\mathrm {d}x_{i+1}}{(x_{i+1} - y_{i})^{1-\alpha +\varepsilon }}, \end{aligned}$$

and the analogous ones without the integration over $y_{i-1}$ and/or $x_{i+1}$. The finiteness of such integrals is easily checked if $\varepsilon > 0$ is small (so that $1-\alpha +\varepsilon < 1$ and $1+\alpha +\varepsilon < 2$).$\square$

## Appendix B: Renewal estimates

In this section we show that assumption (1.7) holds true for renewal processes satisfying (1.1), under either of the following assumptions:

- $\alpha > 1$ (in particular, the renewal process has finite mean);
- the renewal process is generated by a Bessel-like Markov chain, in the spirit of [2].

Actually, it can be shown that condition (1.7) is satisfied in much greater generality, e.g. when one has an analogue of (1.7), but with $u(\cdot )$ replaced by the renewal kernel $K(\cdot )$. This renewal-theoretic framework falls out of the scope of the present work and will be treated elsewhere.

Let us, now, verify (1.7) in the two cases, mentioned above. Note that the precise value of $\varepsilon > 0$ therein is immaterial: if $\varepsilon 'n \le \ell \le \varepsilon n$ for $0 < \varepsilon ' < \varepsilon$, relation (1.7) is always satisfied (with $\delta = 1$), as it follows by (2.10). We then set $\varepsilon = \frac{1}{4}$ for simplicity and rewrite (1.7) as

4.27 $$\begin{aligned} \exists C, n_0 \in (0,\infty ), \ \delta \in (0,1]: \qquad \bigg | \frac{u(n+\ell )}{u(n)} - 1 \bigg | \le C \bigg (\frac{\ell }{n}\bigg )^\delta \quad \ \forall n \ge n_0, \ 0 \le \ell \le \frac{n}{4}. \end{aligned}$$

### **Lemma B.1**

Let $\tau$ be a non-terminating renewal process satisfying (1.1), with $\alpha >1$. Then (4.27) holds true.

### *Proof*

In the case $\alpha >1$, by (1.1) we have $\mathrm E[\tau _1^\eta ]<\infty$ for some $\eta > 1$. We can then apply the following result of Rogozin [28]:

$$\begin{aligned} u(n) = \mathrm P(n\in \tau ) = \frac{1}{\mathrm E[\tau _1]} + \frac{1}{\mathrm E[\tau _1]^2} \sum _{k=n+1}^\infty \mathrm P(\tau _1> k) + R_n, \end{aligned}$$

where $R_n=o(n^{-2(\eta -1)})$ if $\eta \in (1,2)$, and $R_n=o(n^{-\eta })$ if $\eta \ge 2$, as $n\rightarrow \infty$. Note that, for any $\alpha ' \in (1, \alpha )$, by (1.1) we can choose $C$ such that for all $k\in {\mathbb {N}}_0$

$$\begin{aligned} \mathrm P(\tau _1 > k) = \sum _{n=k+1}^\infty \frac{L(n)}{n^{1+\alpha }} \le \sum _{n=k+1}^\infty \frac{C}{n^{1+\alpha '}} \le \frac{C'}{k^{\alpha '}}. \end{aligned}$$

It follows that for $n\in {\mathbb {N}}$ large enough and $0 \le \ell \le \frac{n}{4}$

$$\begin{aligned} \bigg | \frac{u(n+\ell )}{u(n)} - 1 \bigg |&= \bigg | \frac{u(n) - u(n+\ell )}{u(n)} \bigg |\\&\le C \left( \sum _{k=n+1}^{n+\ell } \mathrm P(\tau _1> k) + \frac{1}{n^{2(\eta -1)}} + \frac{1}{(n+\ell )^{2(\eta -1)}}\right) \\&\le C' \Big ( \frac{\ell }{n^{\alpha '}} + \frac{1}{n^{2(\eta -1)}}\Big ) \le C''\Big (\frac{\ell }{n}\Big )^{1 \wedge (2\eta -2)}. \end{aligned}$$

This establishes (4.27) with $\delta =1 \wedge (2\eta -2)$.$\square$

### **Lemma B.2**

Let $\tau$ be a non-terminating renewal process satisfying (1.1), with $\alpha \in (0,1)$, such that $2\tau _1$ has the same distribution as the first return to zero of a nearest-neighbor Markov chain on ${\mathbb {N}}_0$ with $\pm 1$ increments (Remark 1.2). Then (4.27) holds true.

### *Proof*

Let $X$ and $Y$ be two copies of such a Markov chain, starting at the origin at times $-2\ell$ and $0$ respectively, so that

$$\begin{aligned} u(n+\ell ) = \mathrm P(X_{2n}=0) \qquad \hbox {and} \qquad u(n) = \mathrm P(Y_{2n}=0). \end{aligned}$$

(Although $X_n$ is defined for $n \ge -2\ell$, we only look at it for $n \ge 0$.) We can couple $X$ and $Y$ such that they are independent until they meet, at which time they coalesce. Since $X$ and $Y$ are nearest neighbor walks, $X_{2n}=0$ implies $Y_{2n}=0$, and

$$\begin{aligned} 0&\le \mathrm P(Y_{2n}=0) - \mathrm P(X_{2n}=0) = u(n) - u(n+\ell ) \\&= \mathrm P(Y_{2n}=0\ne X_{2n}) = \mathrm P(Y_{2n}=0, X_t\ne 0 \ \forall \ t\in [0, 2n]) \\&\le \mathrm P(Y_{2n}=0) \mathrm P(X_t\ne 0 \ \forall \ t\in [0, 2n]) \ \le \ u(n) \sum _{k=0}^{\ell -1} u(k) \mathrm P(\tau _1>n+\ell -k) \\&\le u(n) \mathrm P(\tau _1>n) \sum _{k=0}^{\ell -1} u(k), \end{aligned}$$

where by the properties of regularly varying functions, see [8, Prop. 1.5.8 and 1.5.10],

4.28 $$\begin{aligned} \mathrm P(\tau _1 >n)&= \sum _{k=n+1}^\infty \frac{L(k)}{k^{1+\alpha }} \sim \frac{L(n)}{\alpha n^\alpha } \qquad \hbox {as } n\rightarrow \infty , \nonumber \\ \sum _{k=0}^{\ell -1} u(k)&= \sum _{k=0}^{\ell -1} \frac{C_\alpha (1+o(1))}{L(k) k^{1-\alpha }} \sim \frac{C_\alpha \ell ^\alpha }{\alpha L(\ell )} \quad \hbox {as } \ell \rightarrow \infty . \end{aligned}$$

Observe that, for every $\varepsilon ' > 0$ there exists $n_0 < \infty$ such that $L(n)/L(\ell ) \le (n/\ell )^{\varepsilon '}$ for $n \ge n_0$ and $\ell \le \frac{n}{4}$, by Potter bounds [8, Theorem 1.5.6], hence

$$\begin{aligned} \frac{u(n) - u(n+\ell )}{u(n)} \le C \, \frac{L(n)}{L(\ell )} \, \bigg (\frac{\ell }{n}\bigg )^\alpha \le C \, \bigg (\frac{\ell }{n}\bigg )^{\alpha -\varepsilon '}, \end{aligned}$$

and therefore (4.27) holds true for any $\delta <\alpha$.$\square$

## Appendix C: An integral estimate

### **Lemma C.1**

Let $\chi \in [0,1)$. Then there exist $C_1, C_2>0$ such that for all $k\in {\mathbb N}$,

4.29 $$\begin{aligned} \idotsint \limits _{0<t_1<\cdots <t_k<1} \frac{\mathrm {d}t_1\cdots \mathrm {d}t_k}{t_1^{\chi }\cdots (t_k-t_{k-1})^{\chi }(1-t_k)^{\chi }} \le C_1 e^{-C_2k\log k}. \end{aligned}$$

Furthermore, for any $v\in (0,1)$ and $k_1, k_2\in {\mathbb {N}}_0:={\mathbb {N}}\cup \{0\}$ with $k:=k_1+k_2\ge 1$,

4.30 $$\begin{aligned} \mathop {\mathop {\idotsint }\limits _{0<t_1<\cdots t_{k_1}<v}}\limits _{v<t_{k_1+1}<\cdots <t_{k}<1} \frac{\mathrm {d}t_1\cdots \mathrm {d}t_{k}}{t_1^{\chi }(t_2-t_1)^{\chi }\cdots (1-t_{k})^{\chi }} \le C_1 e^{-C_2 k\log k}\ v^{(1-\chi )k_1} (1-v)^{(1-\chi )k_2}. \end{aligned}$$

### *Proof*

By the definition of the Dirichlet distribution

$$\begin{aligned} \idotsint \limits _{0<t_1<\cdots <t_k<1} \frac{\mathrm {d}t_1\cdots \mathrm {d}t_k}{t_1^{\chi }\cdots (t_k-t_{k-1})^{\chi }(1-t_k)^{\chi }} =\frac{(\Gamma (1-\chi ))^{k+1}}{\Gamma \big ((k+1)(1-\chi )\big )}. \end{aligned}$$

The bound (4.29) then follows from the properties of the gamma function $\Gamma (\cdot )$.

If $k_1=0$, then (4.30) can be obtained from (4.29) noting that $1/t_1^{\chi } \le (1-v)^\chi /(t_1-v)^{\chi }$ and performing a change of variable. The same applies to the case $k_2=0$. If $k_1, k_2\ge 1$, then denoting the integral in (4.29) by $A_k$, we have

$$\begin{aligned}&\mathop {\mathop {\idotsint }\limits _{0<t_1<\cdots t_{k_1}<v}}\limits _{v<t_{k_1+1}<\cdots <t_{k_1+k_2}<1} \frac{\mathrm {d}t_1\cdots \mathrm {d}t_{k_1+k_2}}{t_1^{\chi }(t_2-t_1)^{\chi }\cdots (1-t_{k_1+k_2})^{\chi }} \\&\qquad = \mathop {\mathop {\iint }\limits _{0<t_{k_1}<v}}\limits _ {v<t_{k_1+1}<1} \frac{\mathrm {d}t_{k_1} \mathrm {d}t_{k_1+1}}{(t_{k_1+1}-t_{k_1})^{\chi }}\ A_{k_1-1} t_{k_1}^{k_1(1-\chi )-1} A_{k_2-1} (1-t_{k_1+1})^{k_2(1-\chi )-1} \\&\qquad \le \ \ C_1^2 e^{-C_2(k_1-1)\log (k_1-1)}e^{-C_2(k_2-1)\log (k_2-1)} \!\!\!\!\!\!\!\!\!\!\!\!\!\! \iint \limits _{0<t_{k_1}<v<t_{k_1+1}<1} \!\!\!\!\!\!\!\!\!\!\!\! \frac{t_{k_1}^{k_1(1-\chi )-1}(1-t_{k_1+1})^{k_2(1-\chi )-1}}{(t_{k_1+1}-t_{k_1})^{\chi }} \mathrm {d}t_{k_1} \mathrm {d}t_{k_1+1} \\&\qquad \le \ \ C_3 e^{-C_4(k_1+k_2)\log (k_1+k_2)}\ v^{(1-\chi )k_1} (1-v)^{(1-\chi )k_2}, \end{aligned}$$

where the last inequality is obtained by first noting that $\max \{k_1, k_2\}\ge (k_1+k_2)/2$, and then replacing $t_{k_1+1}-t_{k_1}$ with $t_{k_1+1}-v$ (resp. $v-t_{k_1}$) if $v<1/2$ (resp. $v\ge 1/2$). In this way the integral factorizes and one obtains (4.30) after adjusting the values of $C_1$ and $C_2$.$\square$

## Footnotes

1 Actually [31] considers the non-conditioned case (1.4), but it can be adapted to the conditioned case.

2 Alternatively, one can write down explicitly the f.d.d. of $(\varvec{X}_t)_{t\in [0,T]}$ in terms of the continuum partition functions $\varvec{Z}^{\alpha ; W, \mathrm c}_{\hat{\beta }, \hat{h}}(s,t)$ (see Sect. 2). We skip the details for the sake of brevity.

3 The presence of $\mathtt g_{\frac{1}{2^n}}(\varvec{\tau })$ and $\mathtt d_{1-\frac{1}{2^n}}(\varvec{\tau })$ in (4.4) is due to the fact that $0$ and $T$ are accumulation points of $\varvec{\tau }$, $\varvec{\mathrm {P}}^{\alpha ; \mathrm c}_1(\mathrm {d}\varvec{\tau })$-a.s., hence $\varvec{\tau }\cap (0,\frac{1}{2^n}] \ne \emptyset$ and $\varvec{\tau }\cap (1-\frac{1}{2^n}, 1] \ne \emptyset$.

## References

[1] Alexander KS (2008). The effect of disorder on polymer depinning transitions. Comm. Math. Phys..

[2] Alexander KS (2011). Excursions and local limit theorems for Bessel-like random walks. Electron. J. Probab..

[3] Alberts T, Khanin K, Quastel J (2014). The intermediate disorder regime for directed polymers in dimension $$1+1$$. Ann. Probab..

[4] Alberts T, Khanin K, Quastel J (2014). The continuum directed random polymer. J. Stat. Phys..

[5] Bertoin, J.: Lévy Processes. Cambridge Tracts in Mathematics, 121. Cambridge University Press, Cambridge (1996)

[6] Billingsley P (1999). Convergence of probability measures.

[7] Berger Q, Caravenna F, Poisat J, Sun R, Zygouras N (2014). The critical curves of the random pinning and copolymer models at weak coupling. Commun. Math. Phys..

[8] Bingham, N.H., Goldie, C.M., Teugels, J.L.: Regular variation. Encyclopedia of Mathematics and its Applications, 27. Cambridge University Press, Cambridge (1987)

[9] Bolthausen E (1989). A note on diffusion of directed polymers in a random environment. Commun. Math. Phys..

[10] Caravenna, F., Sun, R., Zygouras, N.: Polynomial chaos and scaling limits of disordered systems. J. Eur. Math. Soc. arXiv:1312.3357 (to appear) (2013)

[11] Comets F, Yoshida N (2006). Directed polymers in random environment are diffusive at weak disorder. Ann. Probab..

[12] Doney RA (1997). One-sided local large deviation and renewal theorems in the case of infinite mean. Probab. Theory Rel. Fields.

[13] den Hollander, F.: Random polymers. Lectures from the 37th Probability Summer School held in Saint-Flour, 2007. Springer, Berlin (2009)

[14] Ethier SN, Kurtz TG (1986). Markov processes.

[15] Fell JMG (1962). A Hausdorff topology for the closed subsets of a locally compact non-Hausdorff space. Proc. Am. Math. Soc..

[16] Fitzsimmons PJ, Fristedt B, Maisonneuve B (1985). Intersections and limits of regenerative sets. Z. Wahrsch. Verw. Gebiete.

[17] Garsia, A.M.: Continuity properties of Gaussian processes with multidimensional time parameter. Proceedings of the Sixth Berkeley Symposium on Mathematical Statistics and Probability, Vol. II: Probability theory, 369–374. Univ. California Press, Berkeley, Calif., (1972)

[18] Garsia A, Lamperti J (1963). A discrete renewal theorem with infinite mean. Comm. Math. Helv..

[19] Giacomin G (2007). Random polymer models.

[20] Giacomin, G.: Disorder and critical phenomena through basic probability models. Lecture notes from the 40th Probability Summer School held in Saint-Flour, 2010. Springer, Berlin (2011)

[21] Jacod J, Shiryaev AN (2003). Limit theorems for stochastic processes.

[22] Janson, S.: Gaussian Hilbert spaces. vol. 129 of Cambridge Tracts in Mathematics. Cambridge University Press, Cambridge (1997)

[23] Kallenberg O (1997). Foundations of modern probability.

[24] Matheron G (1975). Random sets and integral geometry.

[25] Molchanov I (2005). Theory of random sets.

[26] Mossel E, O’Donnell R, Oleszkiewicz K (2010). Noise stability of functions with low influences: variance and optimality. Ann. Math..

[27] Revuz D, Yor M (1999). Continuous martingales and Brownian motion.

[28] Rogozin BA (1973). An estimate of the remainder term in limit theorems of renewal theory. Teor. Verojatnost. i Primenen..

[29] Roynette, B., Vallois, P., Yor, M.: Penalizing a BES(d) process with $$(0<d<=2)$$ a function of its local time, V. Stud. Sci. Math. Hung. **45**, 67–124 (2008)

[30] Schertzer, E., Sun, R., Swart J.M.: Stochastic flows in the Brownian web and net. Memoirs of the American Mathematical Society 227:1065 (2014)

[31] Sohier J (2009). Finite size scaling for homogeneous pinning models. Alea.

